# What are the perceptions and concerns of people living with diabetes and National Health Service staff around the potential implementation of AI‐assisted screening for diabetic eye disease?

**DOI:** 10.1111/dme.70165

**Published:** 2025-11-10

**Authors:** Kathryn Willis, Royce Shakespeare, Lakshmi Chandrasekaran, Umar Chaudhry, Charlotte Wahlich, Ryan Chambers, Louis Bolter, John Anderson, Abraham Olvera‐Barrios, Jiri Fajtl, Roshan Welikala, Sarah Barman, Samantha Mann, Peter Scanlon, Maged S. Habib, Catherine A. Egan, Adnan Tufail, Christopher G. Owen, Alicja R. Rudnicka, the ARIAS Research Group, John Anderson, Sarah Barman, Louis Bolter, Tom Broad, Ryan Chambers, Lakshmi Chandrasekaran, Umar Chaudhry, Miu Chi Tang, Clare Connor, Karen Easy, Cathy Egan, Davies Eju‐Konem, Jiri Fajtl, Maged Habib, Julie Hapeshi, Aaron Lee, Laura Lodge, Fiona Martin, Samantha Mann, Peter Mitchell, Abdul Mulla Gbenga Olasehinde, Abraham Olvera‐Barrios, Christopher G Owen, Ahmed Patel, Alicja R Rudnicka, Peter Scanlon, Adam Stott, Adnan Tufail, Charlotte Wahlich, Laura Webster, Roshan Welikala, Kathryn Willis

**Affiliations:** ^1^ School of Health and Medical Sciences, Department of Population Health and Policy, City St George's University of London London UK; ^2^ Homerton Healthcare NHS Foundation Trust London UK; ^3^ NIHR Biomedical Research Centre, Moorfields Eye Hospital NHS Foundation Trust and Institute of Ophthalmology University College London London UK; ^4^ School of Computer Science and Mathematics Kingston University London UK; ^5^ Guy's and St Thomas's NHS Foundation Trust London UK; ^6^ Gloucestershire Hospitals NHS Foundation Trust Cheltenham UK; ^7^ South Tyneside and Sunderland NHS Foundation Trust South Shields UK

**Keywords:** artificial intelligence, diabetes, screening, survey, technology

## Abstract

**Aims:**

To explore attitudes of people living with diabetes (PLD) and healthcare professionals (HCP) towards the use of automated retinal image analysis systems using artificial intelligence (AI) in NHS Diabetic Eye Screening Programmes (DESP) and how these perceptions vary by sociodemographic subgroups.

**Methods:**

Two anonymous online surveys (28 questions for PLD and 21 for HCP) were developed to assess attitudes towards AI. Data were collected from four English DESPs, diabetes charities and patient groups between September and December 2023. Likert‐scale responses were analysed using regression to examine subgroup differences.

**Results:**

A total of 1577 PLD and 262 HCP participated. Fifty‐eight per cent of PLD believed AI would perform equally well in all subgroups, compared with 32% of HCP. Seventy‐one per cent of HCP disagreed that AI could replace human grading, and 81% of PLD felt humans should remain responsible for screening outcomes. Both groups supported AI's efficiency but had concerns about data security, trust, job security and who would be responsible for AI errors. Linear regression of Likert scores showed women were less accepting of AI; PLD of Black and Asian ethnicities were more cautious of data security and impact on screening experience. HCP of Asian ethnicity generally held more negative views across themes. Those using more online applications had more positive views towards AI.

**Conclusions:**

While both PLD and HCP recognise AI's potential benefits, concerns regarding security, job impact and errors highlight the need for targeted outreach based on sociodemographic factors.

**What's new?**

**What is already known?**

- The English NHS Diabetic Eye Screening Programme generates approximately 13 million retinal images annually, requiring human grading for diabetic retinopathy.
- AI‐based retinal image analysis systems can detect sight‐threatening diabetic retinopathy as effectively as human graders.

**What this study has found?**

- PLD and HCP acknowledge the potential advantages of AI in DESP but expressed concerns about data security, accountability, safety, job role impacts and AI‐related errors.

**What are the implications of the study?**

- Individual sociodemographic characteristics influenced attitudes towards AI acceptance in diabetic eye screening and should be considered when developing outreach material for translation into practice.

## 1 INTRODUCTION

The UK government and healthcare organisations are increasingly interested in implementing artificial intelligence (AI)‐based software into healthcare services^1^, ^2^, ^3^, ^4^ to increase efficiency and to assist with diagnostics.^5^ There has been heightened interest in using Automated Retinal Image Analysis Systems (ARIAS), including AI, in the English NHS Diabetic Eye Screening Programme (DESP) to assist with the detection of diabetic eye disease, known as diabetic retinopathy (DR). The primary aim of this national programme is to prevent sight‐threatening DR (STDR).^6^, ^7^, ^8^ However, this presents a challenge, given the rising prevalence of diabetes in the United Kingdom,^9^, ^10^ placing greater demand on screening services.^11^, ^12^, ^13^ The NHS DESP generates approximately 13 million retinal images annually,^14^ which are reviewed by up to three trained human graders for the presence and severity of DR. Those with STDR are referred to hospital eye services for further assessment and treatment. This is labour intensive, costly and requires ongoing training and quality assurance of human graders.^15^

We have shown that ARIAS could provide a safe, cost‐effective alternative to a fully human‐led assessment, which could help alleviate service pressures by substantially increasing image‐grading capacity.^15^, ^16^, ^17^ However, to prevent disengagement, prior to implementation, it is important to (i) understand how people living with diabetes (PLD) and NHS staff would receive this technology and (ii) seek views from different age and ethnic groups, particularly as diabetes disproportionately affects people from non‐White backgrounds, who are often diagnosed at younger ages.^18^

Issues raised in existing evidence on opinions of AI in healthcare include public confidence and acceptability, trust, regulation, relationship with humans and risks versus benefits of using AI.^1^, ^19^, ^20^, ^21^, ^22^, ^23^, ^24^, ^25^ For healthcare professionals (HCP), use of AI in healthcare highlighted common themes of trust, governance, efficiency, training and job security.^1^, ^26^, ^27^, ^28^, ^29^, ^30^ These themes were also elicited during the co‐design phase of our survey.^31^ This study presents the quantitative findings of two co‐designed surveys for PLD and HCP, gauging attitudes and perceptions to the potential introduction of AI into the DESP and compares responses between population subgroups.

## 2 METHODS

Our methodology has been published.^31^ In brief, two online surveys were co‐designed using focus group sessions with PLD and HCP, and validated through interviews. Survey questions were co‐designed with PLD and HCP through iterative focus groups and validated via cognitive interviewing to ensure consistent interpretation across participant groups. Most questions about views/perceptions adopted a five‐point Likert‐scale: ‘strongly agree’, ‘agree’, ‘neither agree nor disagree’, ‘disagree’ and ‘strongly disagree’. Both surveys featured positively and negatively framed questions. Most questions were categorised into key themes (Box 1). We specified in survey framing that AI was described as an assistive triage tool rather than a full replacement for human graders. The item on benchmarking of AI outputs was pre‐specified based on concerns raised during co‐design workshops, where participants highlighted the need to understand how AI performance compared to established standards.

BOX 1 **Key survey themes identified.**

**Table:** 

People living with diabetes (PLD)	Healthcare professionals (HCP)
Demographic questions Technology in your daily life General questions about AI for eye screening Efficiency Data responsibility/security Trust Screening experience	Demographic questions Technology in your daily life General questions about AI for eye screening Efficiency Data regulation/security Trust Impact on workforce Screening experience & patient‐practitioner relationship

### 2.1 Sample representativeness

#### 2.1.1 People living with diabetes cohort

The participating NHS DESPs serve a diverse population of sociodemographic groups.^31^ Additional responses were sought via charities including Diabetes UK and Diabetes Health and Wellness Foundation, and patient groups.

#### 2.1.2 Healthcare professional cohort

HCP currently working in the NHS DESP were eligible to participate, and were targeted through participating DESPs with the addition of South Tyneside DESP and British Association of Retinal Screeners (BARS).

### 2.2 Survey distribution

The surveys were hosted on Jisc Online Surveys, and were open from 1 September 2023 until 31 December 2023. The PLD survey was distributed via text message (NEL and SEL DESPs), and via a push‐to‐web approach by Gloucestershire DESP with handouts provided by screeners. PLD representatives were given a unique URL to share with patient groups to facilitate further recruitment. Charities shared the survey link via their websites. The HCP survey link was distributed by the DESP clinical lead or programme manager via email. BARS contacted their mailing list and hosted the survey link on their website/newsletters.

### 2.3 Sample size

Sample size was based on detecting one‐step mean differences in Likert scores by population subgroups with ~90% power and alpha set to 0.05^31^ (see Text S1) and inflated to allow for 1% response rates.

### 2.4 Statistical analysis

Analyses were conducted using Stata SE V18 (StataCorp LLC, TX, USA). The HCP and PLD surveys were analysed separately. Five‐point Likert‐scale responses were used from −2 to +2 to represent ‘strongly disagree’ to ‘strongly agree’ responses. Responses of ‘strongly agree’ and ‘agree’ were combined and referred to as agreement; ‘strongly disagree’ and ‘disagree’ as disagreement. Likert‐scale question scores within a theme were also combined (see Box 1) at an individual level to give a mean score per theme. A positive score indicated an overall positive view/perception for a given question/theme, whereas a negative score indicated a negative view or degree of concern. Scores are presented as means with 95% confidence intervals.

Multivariable linear regression was used to examine statistically significant heterogeneity in theme scores (Box 1) by population subgroup. For the PLD survey, models were adjusted for site, age group, sex, ethnicity, educational qualification, employment status, Townsend score, diabetes type, duration of diabetes, DESP attendance, number of applications used in daily life and use of health‐based apps. Diabetes type was included as a covariate to account for differences in age of onset, disease trajectory and care experiences, which may plausibly influence attitudes towards AI in screening. HCP survey analyses were adjusted for site, age group, sex, ethnicity, Townsend score, occupational role, length in current role, number of online applications used in daily life and use of health‐based apps. The base group for regression analyses was selected based on the largest sample size. Interpretation of adjusted Likert score differences focused on the magnitude of statistically significant differences (*p* < 0.05).

For standalone questions, multiple logistic regression was used to determine the odds of agreement with statements (‘strongly agree’ and ‘agree’ combined) using the same adjustments. Sensitivity analyses restricted the regression models to the subset of HCP and PLD who used health‐based apps to assess impact on subgroup differences.

### 2.5 Manuscript patient professional involvement

Preliminary findings and manuscript drafts were shared with focus group participants for feedback. After focus group sessions, PLD and HCP were asked to identify the most important messages from a PLD and HCP perspective, were AI to be incorporated into the NHS DESP. Received feedback was incorporated into the manuscript.

Focus group participants were invited to be named as part of the ARIAS Research Group. Ethical approval was obtained by the NHS Research Ethics Committee (IRAS ID: 316631).

## 3 RESULTS

In total, 32,083 text messages were sent to PLD across the NEL and SEL DESP, resulting in 1532 responses (rate: 4.8%). Additionally, 1000 handouts were distributed at Gloucestershire DESP, yielding 35 responses (rate: 3.5%), and there were 12 responses via charities or the PPI group sharing the survey link (Figure S1). Overall 1577 PLD responded to the survey.

Table 1 outlines the demographic characteristics of PLD respondents. Most were men, aged 60–69, and either retired or employed full‐time. 64% identified as White. 82% of respondents had Type 2 diabetes, with about a third diagnosed for less than 5 years and 40% living with diabetes for 6–16 years.

**TABLE 1:** People living with diabetes respondents' characteristics by recruitment centre.

Characteristic	NEL	SEL	Gloucester	Other	Total
Number	558	972	35	12	1577
Median age (min, max) (years)	62.5 (16, 100)	64 (17, 100)	61 (20, 73)	48 (24, 75)	63 (16, 100)
Age group					
<40	26 (4.7)	35 (3.6)	2 (5.7)	3 (25.0)	66 (4.2)
≥40 to <50	64 (11.5)	73 (7.5)	4 (11.4)	4 (33.3)	145 (9.2)
≥50 to <60	126 (22.6)	189 (19.4)	7 (20.0)	4 (33.3)	326 (20.7)
≥60 to <70	191 (34.2)	337 (34.7)	17 (48.6)	0 (0.0)	545 (34.6)
≥70 to ≤100	151 (27.1)	292 (30.0)	5 (14.3)	1 (8.3)	449 (28.5)
Missing	0 (0.0)	46 (4.7)	0 (0.0)	0 (0.0)	46 (2.9)
Woman (%)	193 (34.6)	396 (40.7)	10 (28.6)	10 (83.3)	609 (38.6)
Ethnicity					
White	284 (50.9)	679 (69.9)	33 (94.3)	11 (91.7)	1007 (63.9)
Black	82 (14.7)	162 (16.7)	0 (0.0)	0 (0.0)	244 (15.5)
Asian	163 (29.2)	67 (6.9)	2 (5.7)	0 (0.0)	232 (14.7)
Mixed/other/prefer not to say	29 (5.2)	64 (6.6)	0 (0.0)	1 (8.3)	94 (6.0)
Highest level of qualification					
No qualification/GCSE level	176 (31.5)	307 (31.6)	11 (31.4)	2 (16.7)	496 (31.5)
A level or equivalent/other higher education below degree level	152 (27.2)	218 (22.4)	8 (22.9)	2 (16.7)	380 (24.1)
Degree/degree‐level vocational qualification or above	174 (31.2)	381 (39.2)	15 (42.9)	7 (58.3)	577 (36.6)
Prefer not to say	51 (9.1)	57 (5.9)	1 (2.9)	1 (8.3)	110 (7.0)
Other	5 (0.9)	9 (0.9)	0 (0.0)	0 (0.0)	14 (0.9)
Employment status					
Full‐time employment	181 (32.4)	284 (29.2)	19 (54.3)	6 (50.0)	490 (31.1)
Part time employment	49 (8.8)	84 (8.6)	3 (8.6)	1 (8.3)	137 (8.7)
Not working (seeking work, looking after home/family, student, sickness/disability)	75 (13.4)	113 (11.6)	1 (2.9)	3 (25.0)	192 (12.2)
Retired	219 (39.2)	453 (46.6)	12 (34.3)	1 (8.3)	685 (43.4)
Prefer not to say/other	34 (6.1)	38 (3.9)	0 (0.0)	1 (8.3)	73 (4.6)
Townsend score					
≤−0.5 (least deprived)	49 (8.8)	209 (21.5)	31 (88.6)	3 (25.0)	292 (18.5)
≥−0.5 to ≤2	111 (19.9)	186 (19.1)	1 (2.9)	3 (25.0)	301 (19.1)
>2 to ≤4	90 (16.1)	226 (23.3)	3 (8.6)	0 (0.0)	319 (20.2)
>4 to ≤6	86 (15.4)	195 (20.1)	0 (0.0)	4 (33.3)	285 (18.1)
>6 (most deprived)	222 (39.8)	156 (16.0)	0 (0.0)	2 (16.7)	380 (24.1)
Diabetes type					
Type 1	51 (9.1)	87 (9.0)	6 (17.1)	9 (75.0)	153 (9.7)
Type 2	460 (82.4)	804 (82.7)	27 (77.1)	3 (25.0)	1294 (82.1)
Other	47 (8.4)	81 (8.3)	2 (5.7)	0 (0.0)	130 (8.2)
Duration of diabetes					
0–5 years	208 (37.3)	342 (35.2)	15 (42.9)	2 (16.7)	567 (36.0)
6–10 years	132 (23.7)	234 (24.1)	5 (14.3)	2 (16.7)	373 (23.7)
11–15 years	74 (13.3)	174 (17.9)	6 (17.1)	1 (8.3)	255 (16.2)
16+ years	144 (25.8)	208 (21.4)	9 (25.7)	7 (58.3)	368 (23.3)
Missing	0 (0.0)	14 (1.4)	0 (0.0)	0 (0.0)	14 (0.9)
Last attend DESP for screening					
Within last 2 years	498 (89.2)	846 (87.0)	33 (94.3)	12 (100.0)	1389 (88.1)
NOT within last 2 years (inc. first time attender/do not remember)	60 (10.8)	126 (13.0)	2 (5.7)	0 (0.0)	188 (11.9)
Technology in daily life					
No. of applications used in daily life					
<5	272 (48.7)	392 (40.3)	5 (14.3)	2 (16.7)	671 (42.5)
≥5	286 (51.3)	580 (59.7)	30 (85.7)	10 (83.3)	906 (57.5)
Use of selected applications to answer health‐related questions: % Yes	301 (53.9)	535 (55.0)	22 (62.9)	11 (91.7)	869 (55.1)
Trust in health‐based results from used applications					
Never/rarely/unsure	13 (4.3)	33 (6.2)	4 (18.2)	0 (0.0)	50 (5.8)
Sometimes	148 (49.2)	312 (58.3)	12 (54.5)	6 (54.5)	478 (55.0)
Often	85 (28.2)	127 (23.7)	4 (18.2)	5 (45.5)	221 (25.4)
Very often/always	55 (18.3)	63 (11.8)	2 (9.1)	0 (0.0)	120 (13.8)
Review of images at DESP appointment					
Reviewed by one healthcare practitioner	229 (41.0)	405 (41.7)	24 (68.6)	8 (66.7)	666 (42.2)
Reviewed by two healthcare practitioners	94 (16.8)	192 (19.8)	4 (11.4)	1 (8.3)	291 (18.5)
Only AI technology reviewed the image	4 (0.7)	13 (1.3)	0 (0.0)	0 (0.0)	17 (1.1)
Reviewed by both healthcare practitioner(s) and AI technology	61 (10.9)	99 (10.2)	3 (8.6)	2 (16.7)	165 (10.5)
Don't know	170 (30.5)	263 (27.1)	4 (11.4)	1 (8.3)	438 (27.8)

*Note*: Data are counts (column %) unless otherwise specified.

Abbreviations: NEL, North East London; SEL, South East London.

In total, 262 HCPs responded to the survey. Direct invites from DESP resulted in a 71.4% response rate, with 125 additional responses from BARS (Figure S1). Table 2 outlines the demographic breakdown of HCP respondents. Over two‐thirds were women, and the ethnic distribution was 77% White, 14% Asian and 2.7% Black. The majority (39%) had a mid‐level role in the DESP, and 43% had been working in the DESP for less than 5 years.

**TABLE 2:** Healthcare professional respondents' characteristics by recruitment centre.

	NEL	SEL	Gloucester	ST	BARS	Total
*N*	55	30	30	22	125	262
Median age (min, max) (years)	39 (22, 66)	41 (23, 62)	41 (28,70)	52.5 (26, 65)	46 (23, 72)	43 (22, 72)
Age group						
<30 years	12 (21.8)	4 (13.3)	3 (10.0)	1 (4.5)	14 (11.2)	34 (13.0)
≥30 to <40 years	16 (29.1)	9 (30.0)	9 (30.0)	4 (18.2)	31 (24.8)	69 (26.3)
≥40 to <50 years	14 (25.5)	10 (33.3)	7 (23.3)	4 (18.2)	31 (24.8)	66 (25.2)
≥50 to <60 years	8 (14.5)	6 (20.0)	5 (16.7)	9 (40.9)	33 (26.4)	61 (23.3)
60+ years	5 (9.1)	1 (3.3)	6 (20.0)	4 (18.2)	16 (12.8)	32 (12.2)
Woman (%)	39 (70.9)	22 (73.3)	20 (66.7)	18 (81.8)	82 (65.6)	181 (69.1)
Ethnicity						
White	22 (40.0)	19 (63.3)	27 (90.0)	21 (95.5)	112 (89.6)	201 (76.7)
Black	3 (5.5)	2 (6.7)	1 (3.3)	0 (0.0)	1 (0.8)	7 (2.7)
Asian	21 (38.2)	4 (13.3)	1 (3.3)	0 (0.0)	10 (8.0)	36 (13.7)
Mixed/Other/prefer not to say	9 (16.4)	5 (16.7)	1 (3.3)	1 (4.5)	2 (1.6)	18 (6.9)
Townsend score						
<−2 (least deprived)	3 (5.5)	1 (3.3)	11 (36.7)	0 (0.0)	41 (32.8)	56 (21.4)
≥−2 to <−0.5	2 (3.6)	4 (13.3)	12 (40.0)	10 (45.5)	24 (19.2)	52 (19.8)
≥−0.5 to <2	9 (16.4)	6 (20.0)	1 (3.3)	10 (45.5)	35 (28.0)	61 (23.3)
≥2 to <4	14 (25.5)	4 (13.3)	5 (16.7)	2 (9.1)	14 (11.2)	39 (14.9)
≥4 (most deprived)	27 (49.1)	15 (50.0)	1 (3.3)	0 (0.0)	8 (6.4)	51 (19.5)
Missing	0 (0.0)	0 (0.0)	0 (0.0)	0 (0.0)	3 (2.4)	3 (1.1)
Role						
Clinical lead/management position	12 (21.8)	10 (33.3)	5 (16.7)	3 (13.6)	38 (30.4)	68 (26.0)
Senior screener/grader	19 (34.5)	6 (20.0)	5 (16.7)	3 (13.6)	26 (20.8)	59 (22.5)
Screener/grader/photographer/SLTBe/optometrist	20 (36.4)	10 (33.3)	15 (50.0)	6 (27.3)	51 (40.8)	102 (38.9)
Administrator/IT officer/Fail safe officer/other	4 (7.3)	4 (13.3)	5 (16.7)	10 (45.5)	10 (8.0)	33 (12.6)
Length of role						
<5	19 (34.5)	16 (53.3)	12 (40.0)	11 (50.0)	54 (43.2)	112 (42.7)
≥5 to <10	15 (27.3)	8 (26.7)	8 (26.7)	2 (9.1)	36 (28.8)	69 (26.3)
≥10	21 (38.2)	6 (20.0)	10 (33.3)	9 (40.9)	35 (28.0)	81 (30.9)
Technology in daily life						
No. of applications used in daily life						
<5	8 (14.5)	2 (6.7)	3 (10.0)	6 (27.3)	8 (6.4)	27 (10.3)
≥5	47 (85.5)	28 (93.3)	27 (90.0)	16 (72.7)	117 (93.6)	235 (89.7)
Use selected applications to answer health‐related questions						
% Yes	33 (60.0)	17 (56.7)	17 (56.7)	12 (54.5)	84 (67.2)	163 (62.2)
Trust in health‐based results from used applications						
Never/rarely/unsure	3 (9.1)	2 (11.8)	0 (0.0)	2 (16.7)	8 (9.5)	15 (9.2)
Sometimes	21 (63.6)	6 (35.3)	13 (76.5)	6 (50.0)	45 (53.6)	91 (55.8)
Often	9 (27.3)	7 (41.2)	2 (11.8)	3 (25.0)	26 (31.0)	47 (28.8)
Very often/always	0 (0.0)	2 (11.8)	2 (11.8)	1 (8.3)	5 (6.0)	10 (6.1)

*Note*: Data are counts (column %) unless otherwise specified.

Abbreviations: NEL, North East London; SEL, South East London; ST, South Tyneside; BARS, British Association of Retinal Screeners.

### 3.1 Responses to standalone questions

**FIGURE 1:**
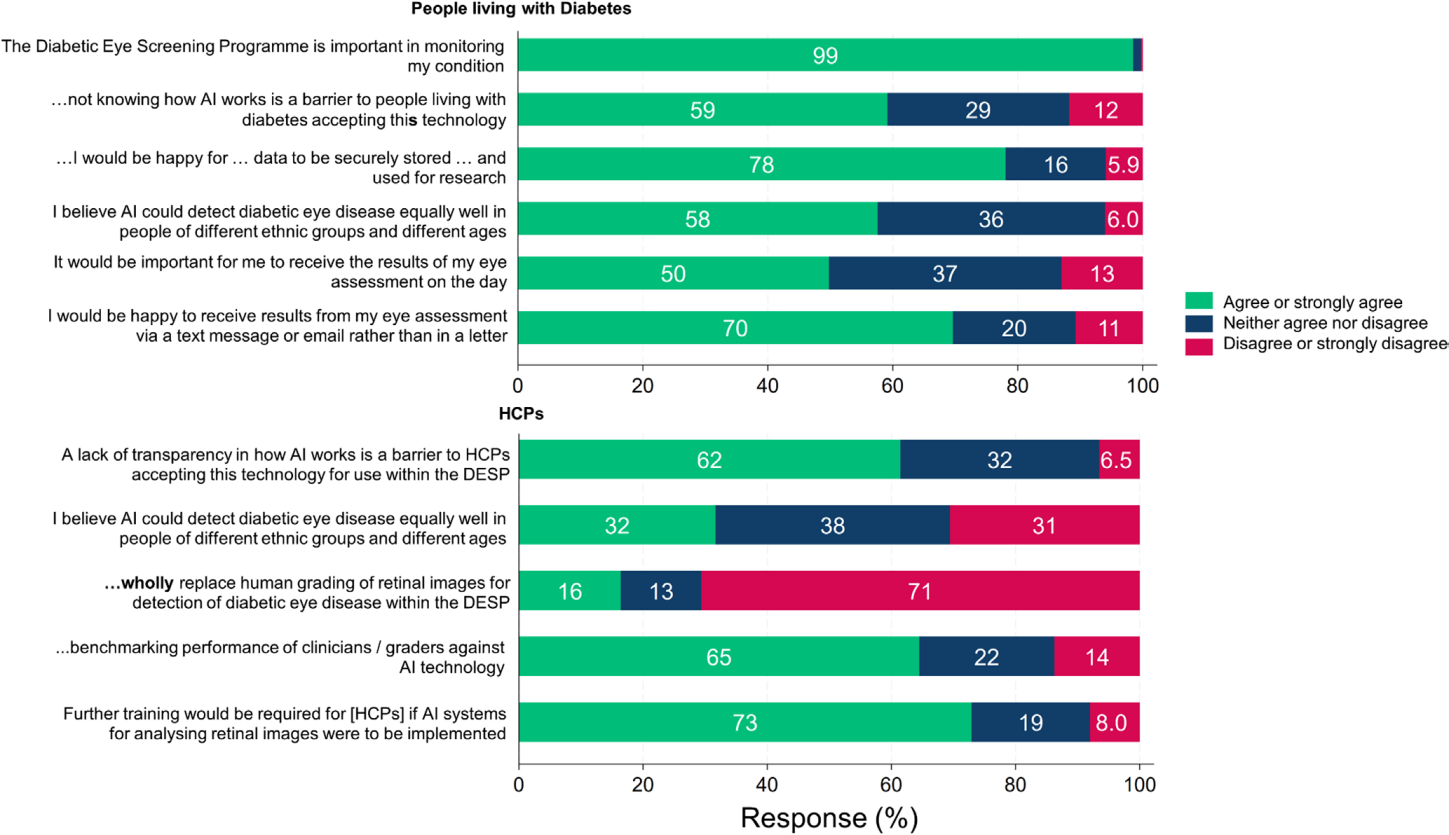
Bar charts of responses from people living with diabetes and healthcare professionals (HCP) to stand alone questions. One HCP standalone question also features a repeat question from the impact on the workforce theme (benchmarking performance of clinicians/graders against AI technology) as this was identified as important question to report individually. Green bars show %strongly agree or agree; Blue bars show %neither agree nor disagree; red bars show %strongly disagree or disagree. Percentage values above 10 are rounded to whole numbers for display purposes and ease of interpretation, and hence may not add up to 100%.

Standalone questions were treated separately from theme‐based questions (Figure 1). Results for each question by site are provided in Tables S1 and S2. Among PLD, 58% agreed that AI could work equally well across different ages and ethnicities, while 36% neither agreed nor disagreed and only 6% disagreed. However, HCPs had more divided views: 32% agreed, 31% disagreed and 38% neither agreed nor disagreed. Regarding AI's transparency, 59% of PLD and 62% of HCPs agreed that not understanding how AI works was a barrier to accepting AI in the DESP. Additionally, 73% of HCPs agreed they would require further training before AI could be implemented, and 65% were concerned that HCP performance might be benchmarked against AI.

For PLD, almost all (99%) agreed that the DESP was important for monitoring their condition. About 78% were comfortable with their diabetic eye screening data being securely stored and used for research, and 70% would prefer to receive results by text or email. Half of PLD agreed that receiving their results on the same day was important.

### 3.2 Likert scores by survey theme

**FIGURE 2:**
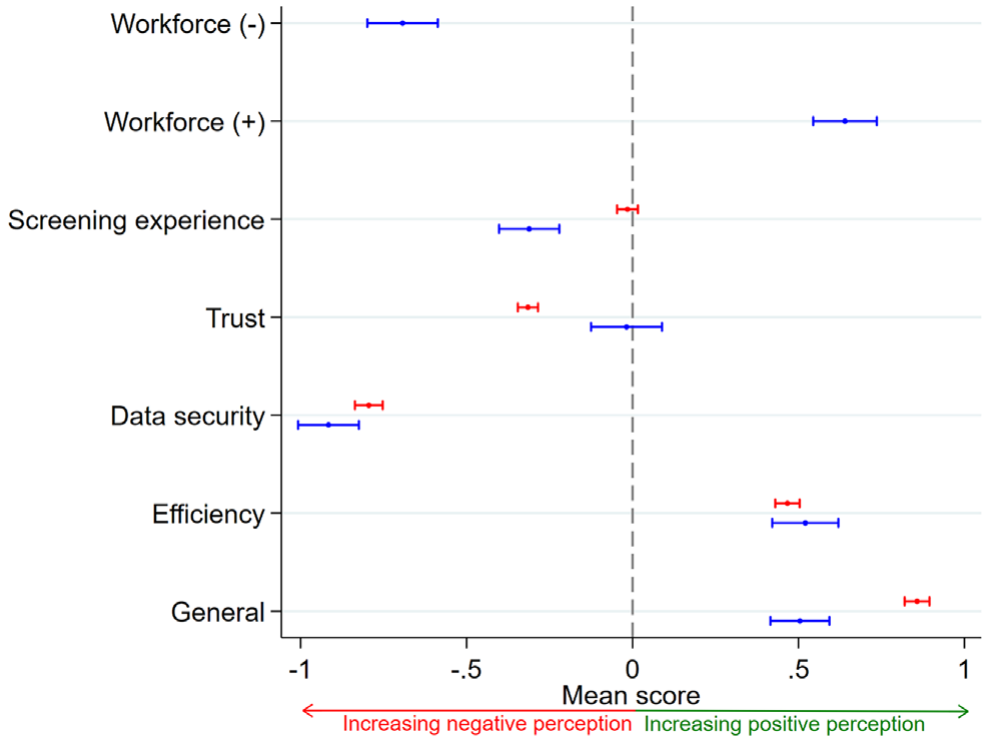
Mean Likert scores by survey themes. Red and blue denotes PLD and HCP, respectively. A score greater than 0 indicates a more positive attitude towards AI in relation to each theme, whereas a score less than 0 indicates a negative attitude towards AI. Error bars are 95% confidence intervals. Workforce (+ve) represents questions which consider potential positives of AI in the DESP and workforce (−ve) represents questions which consider potential negatives of AI in the DESP. (Full list of the survey questions are in supplementary material.)

**FIGURE 3:**
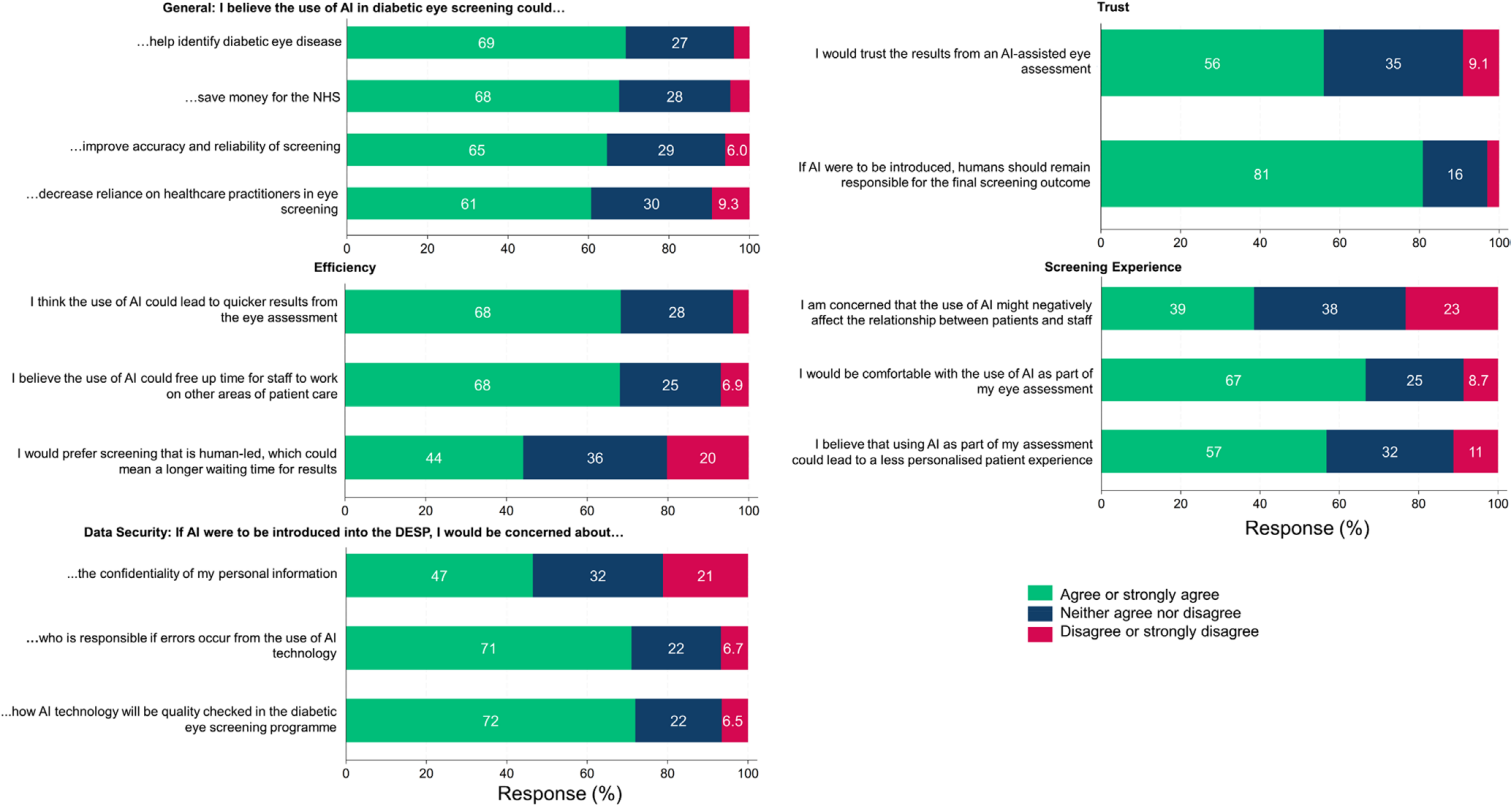
Bar charts of responses from people living with diabetes to individual questions within themes General, Efficiency, Data Security, Trust and Screening Experience. Percentage values above 10 are rounded to whole numbers for display purposes and ease of interpretation, and hence may not add up to 100%.

**FIGURE 4:**
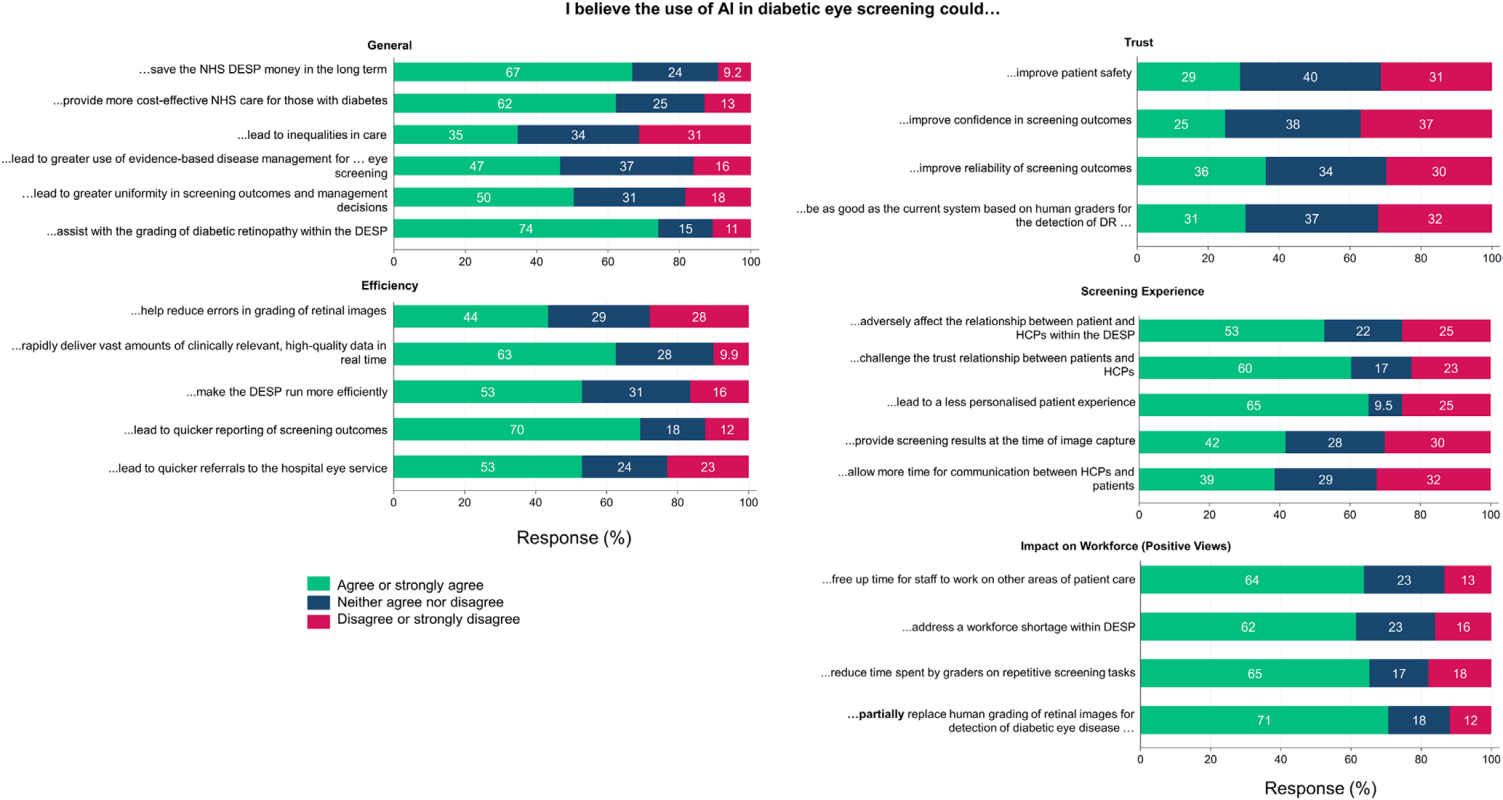
Bar charts of responses from healthcare professionals (HCP) to individual questions within themes of General, Efficiency, Trust, Screening Experience and Impact on the Workforce (positive views). Percentage values above 10 are rounded to whole numbers for display purposes and ease of interpretation, and hence may not add up to 100%.

**FIGURE 5:**
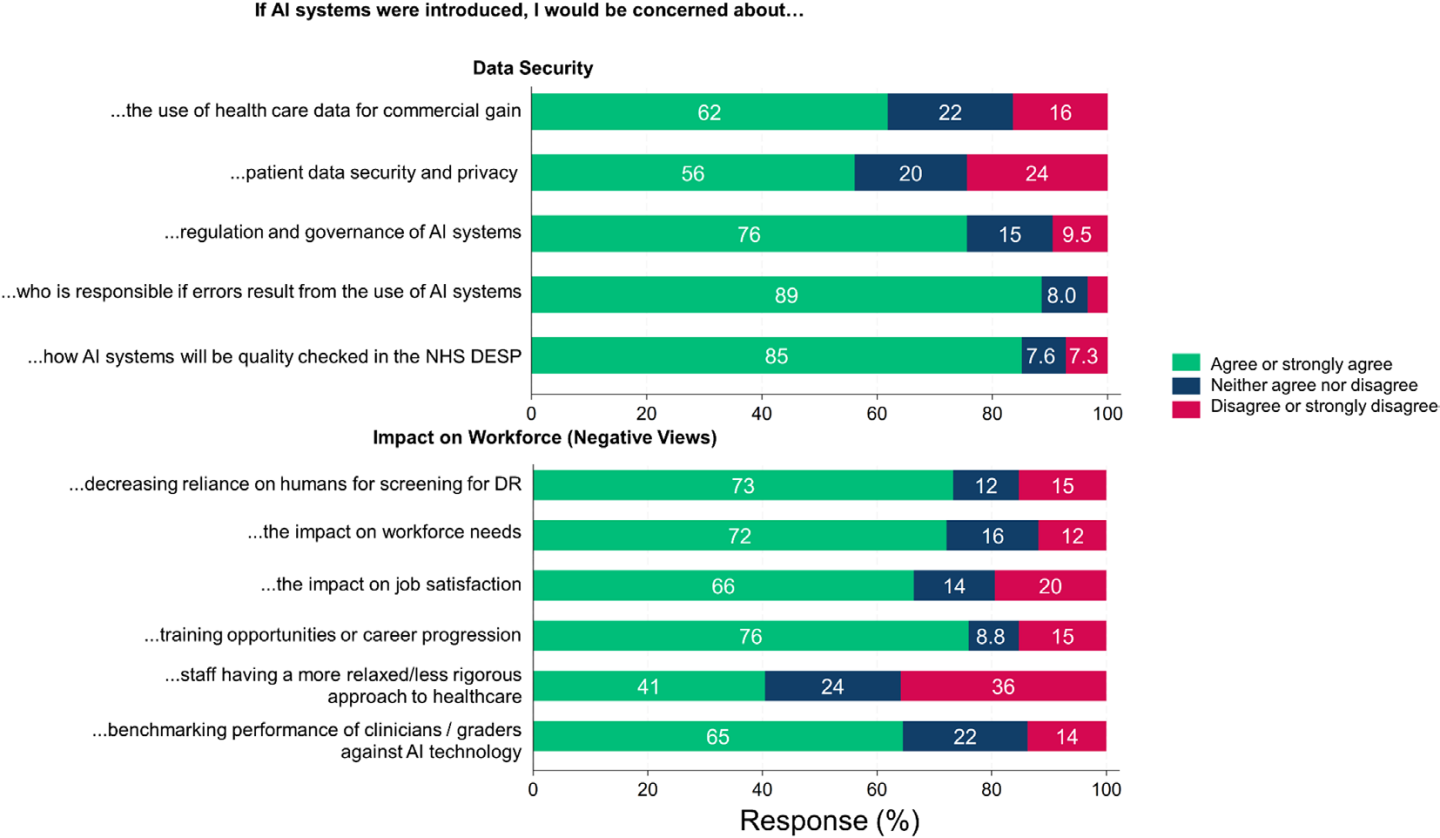
Bar charts of responses from healthcare professionals (HCP) to individual questions within themes of Data Security and Impact on the Workforce (negative views). Percentage values above 10 are rounded to whole numbers for display purposes and ease of interpretation, and hence may not add up to 100%.

Figure 2 shows mean Likert scores by theme for both PLD and HCP. Negative scores indicate concerns about AI, while positive scores reflect a more favourable attitude. Overall, both groups showed a positive attitude towards AI's potential to improve efficiencies, with HCPs recognising its workforce benefits. Both PLD and HCPs shared concerns about data security, but PLD expressed lower trust, while HCPs were concerned about the impact on workforce dynamics and the screening experience (Figures 3, 4, 5).

### 3.3 PLD responses by themes (Figure 3)

#### 3.3.1 General themes

A majority of PLD agreed that AI‐assisted screening could help identify diabetic eye disease, save money for the NHS and improve the accuracy and reliability of screening results. 60% believed AI could decrease reliance on HCPs.

#### 3.3.2 Efficiency

PLD saw the potential for AI to lead to quicker reporting and free up HCP time for other patient care. However, 44% preferred human‐led eye screening even if it meant waiting longer for results.

#### 3.3.3 Data security/responsibility

Over 70% of PLD expressed concerns about the quality assurance of AI in the DESP and who would be responsible for AI errors. Around 47% were concerned about the confidentiality of their personal information.

#### 3.3.4 Trust

81% of PLDs agreed that HCPs should remain responsible for the final screening outcome if AI were incorporated, and 56% would trust AI‐assisted eye assessment results, with, 9% expressing mistrust.

#### 3.3.5 Screening experience

39% of PLD respondents were concerned that AI could harm the HCP‐PLD relationship, and 57% believed it could lead to a less personalised experience. Only 9% were uncomfortable with AI‐assisted assessments (67% reported being comfortable).

#### 3.3.6 Adjusted differences in Likert scores by subgroups of PLD

Table 3 summarises the adjusted differences in Likert scores for various PLD subgroups. In general, women had more negative views than men across all themes. Across ethnic subgroups, Black and Asian PLD were more concerned about data security and the impact on the screening experience. Respondents with lower educational levels expressed more concerns about the screening experience, but had more positive views on trust in AI. This may reflect a misunderstanding about how AI might influence interaction with screening services. Subgroup analysis found no consistent significant differences in attitudes or concerns between people with Type 1 versus Type 2 diabetes. There were no notable differences in attitudes between respondents recruited from different sites/groups.

**TABLE 3:** Multivariable linear regression results for people living with diabetes.

Differences in scores compared to base group (95% confidence interval)					
Characteristics	General score	Efficiency score	Data Security score	Trust score	Screening experience
Site Base: South East London					
North East London	0.03 (−0.06, 0.11)	−0.05 (−0.13, 0.04)	−0.08 (−0.17, 0.02)	0.02 (−0.05, 0.09)	−0.06 (−0.12, 0.01)
Gloucester	0.18 (−0.09, 0.44)	0.16 (−0.10, 0.42)	0.23 (−0.07, 0.52)	0.13 (−0.08, 0.34)	0.21 (0.00, 0.42)*
Other	0.30 (−0.14, 0.73)	0.31 (−0.12, 0.73)	0.24 (−0.25, 0.73)	0.39 (0.03, 0.75)*	0.10 (−0.25, 0.45)
Age group Base: ≥50 to <60 years					
<40 years	0.00 (−0.20, 0.20)	0.03 (−0.17, 0.22)	0.13 (−0.10, 0.35)	0.11 (−0.06, 0.27)	0.00 (−0.16, 0.16)
≥40 to <50 years	0.05 (−0.10, 0.20)	0.03 (−0.12, 0.17)	0.20 (0.03, 0.36)*	0.06 (−0.07, 0.18)	−0.02 (−0.13, 0.10)
≥60 to <70 years	0.11 (−0.01, 0.22)	0.11 (0.00, 0.22)	0.13 (0.00, 0.26)*	0.05 (−0.04, 0.15)	0.03 (−0.06, 0.12)
≥70 to ≤100 years	0.12 (−0.02, 0.26)	0.13 (−0.02, 0.27)	0.07 (−0.09, 0.23)	0.09 (−0.02, 0.21)	0.03 (−0.08, 0.15)
Missing	0.07 (−0.16, 0.31)	−0.23 (−0.46, 0.00)*	−0.11 (−0.38, 0.15)	0.19 (−0.01, 0.38)	−0.16 (−0.34, 0.03)
Sex Base: Male					
Woman	−0.18 (−0.26, −0.10)^ⱡ^	−0.19 (−0.27, −0.11)^ⱡ^	−0.19 (−0.28, −0.10)^ⱡ^	−0.15 (−0.22, −0.09)^ⱡ^	−0.15 (−0.21, −0.09)^ⱡ^
Prefer not to say	−0.01 (−0.46, 0.45)	−0.31 (−0.75, 0.13)	0.27 (−0.24, 0.78)	−0.02 (−0.39, 0.35)	−0.06 (−0.41, 0.30)
Ethnicity					
Base: White					
Black	−0.04 (−0.16, 0.07)	−0.07 (−0.19, 0.04)	−0.13 (−0.26, 0.00)*	0.03 (−0.06, 0.13)	−0.35 (−0.44, −0.25)^ⱡ^
Asian	0.10 (−0.02, 0.22)	−0.02 (−0.13, 0.09)	−0.27 (−0.40, −0.14)^ⱡ^	0.12 (0.02, 0.21)*	−0.26 (−0.35, −0.16)^ⱡ^
Mixed/other/prefer not to say	−0.16 (−0.33, 0.00)*	−0.12 (−0.28, 0.05)	−0.02 (−0.20, 0.17)	−0.03 (−0.17, 0.11)	−0.16 (−0.29, −0.02)*
Highest qualification Base: Degree or equivalent or above					
No qualification or GCSE level qualification	0.07 (−0.03, 0.16)	0.01 (−0.08, 0.11)	0.05 (−0.05, 0.16)	0.15 (0.07, 0.23)^ⱡ^	−0.18 (−0.26, −0.10)^ⱡ^
A level or equivalent higher education qualification	0.00 (−0.10, 0.09)	−0.04 (−0.14, 0.05)	−0.04 (−0.15, 0.07)	0.08 (0.00, 0.16)*	−0.14 (−0.22, −0.06)^ⱡ^
Prefer not to say	−0.16 (−0.32, 0.00)*	−0.22 (−0.38, −0.06)*	−0.15 (−0.33, 0.03)	−0.04 (−0.17, 0.09)	−0.30 (−0.42, −0.17)^ⱡ^
Other	0.07 (−0.32, 0.47)	−0.14 (−0.53, 0.25)	−0.36 (−0.80, 0.09)	−0.01 (−0.34, 0.32)	−0.24 (−0.56, 0.07)
Employment status Base: Retired					
In full‐time employment	0.02 (−0.10, 0.14)	0.03 (−0.09, 0.15)	−0.01 (−0.15, 0.12)	−0.01 (−0.11, 0.09)	0.02 (−0.08, 0.11)
In part‐time employment	0.08 (−0.07, 0.23)	0.05 (−0.10, 0.20)	0.03 (−0.14, 0.20)	0.02 (−0.11, 0.14)	0.05 (−0.07, 0.17)
Not working (seeking work, looking after home/family, student, sickness/disability)	0.05 (−0.09, 0.19)	−0.02 (−0.16, 0.12)	0.00 (−0.16, 0.16)	0.00 (−0.11, 0.12)	−0.02 (−0.14, 0.09)
Prefer not to say/other	−0.06 (−0.26, 0.14)	−0.02 (−0.21, 0.18)	−0.14 (−0.36, 0.09)	−0.06 (−0.22, 0.10)	−0.08 (−0.24, 0.08)
Townsend score: Base: >6					
≤−0.5 (least deprived)	−0.08 (−0.21, 0.05)	−0.08 (−0.20, 0.05)	0.00 (−0.15, 0.14)	−0.12 (−0.22, −0.01)*	−0.03 (−0.13, 0.07)
>−0.5 to ≤2	−0.11 (−0.23, 0.01)	0.01 (−0.11, 0.12)	−0.01 (−0.14, 0.13)	−0.07 (−0.17, 0.03)	−0.05 (−0.14, 0.05)
>2 to ≤4	0.03 (−0.08, 0.15)	0.02 (−0.09, 0.14)	0.02 (−0.11, 0.15)	0.01 (−0.08, 0.11)	0.05 (−0.04, 0.14)
>4 to ≤6 (most deprived)	0.04 (−0.08, 0.16)	0.05 (−0.06, 0.17)	0.02 (−0.11, 0.16)	0.05 (−0.05, 0.14)	0.06 (−0.04, 0.15)
About you					
Diabetes type: Base: Type 2					
Type 1	0.03 (−0.11, 0.17)	0.08 (−0.05, 0.22)	−0.03 (−0.19, 0.12)	0.02 (−0.09, 0.13)	0.04 (−0.07, 0.15)
Other/don't know	0.06 (−0.09, 0.20)	−0.03 (−0.17, 0.11)	0.07 (−0.10, 0.23)	0.03 (−0.08, 0.15)	0.12 (0.00, 0.23)*
Duration of diabetes: Base: 0–5 years					
6–10 years	0.08 (−0.02, 0.19)	0.07 (−0.03, 0.17)	0.00 (−0.12, 0.11)	0.02 (−0.06, 0.11)	0.02 (−0.06, 0.10)
11–15 years	0.20 (0.09, 0.32)^ⱡ^	0.15 (0.03, 0.26)*	0.08 (−0.05, 0.21)	0.07 (−0.03, 0.16)	0.08 (−0.01, 0.18)
16+ years	0.09 (−0.02, 0.20)	0.14 (0.03, 0.24)*	0.05 (−0.07, 0.17)	0.01 (−0.08, 0.10)	0.09 (0.00, 0.18)*
Missing	0.11 (−0.30, 0.53)	0.15 (−0.25, 0.56)	0.07 (−0.39, 0.53)	0.13 (−0.21, 0.46)	−0.14 (−0.47, 0.19)
Last attended DESP: Base: Within last 2 years					
NOT within last 2 years (including first time attender/do not remember/missing)	0.05 (−0.07, 0.17)	0.10 (−0.02, 0.22)	−0.03 (−0.17, 0.11)	0.03 (−0.07, 0.13)	0.03 (−0.07, 0.13)
Technology in daily life: Base: 5 or more applications					
Less than 5 applications	−0.01 (−0.09, 0.07)	−0.09 (−0.17, −0.01)*	−0.11 (−0.20, −0.02)*	0.01 (−0.05, 0.08)	−0.15 (−0.21, −0.09)^ⱡ^
Use of health‐based apps: Base: yes					
No	−0.17 (−0.25, −0.09)^ⱡ^	−0.18 (−0.26, −0.11)^ⱡ^	−0.05 (−0.13, 0.04)	−0.10 (−0.17, −0.04)^†^	−0.11 (−0.17, −0.05)^ⱡ^

* 0.05 ≤ *p*‐value ≥0.01.

^†^ 0.01 > *p*‐value ≥0.001.

^ⱡ^ *p* < 0.001.

### 3.4 HCP responses by themes (Figures 4 and 5)

#### 3.4.1 General

Most HCPs (62%–70%) agreed that AI could save money for the NHS, assist in grading diabetic eye disease and provide cost‐effective care. However, responses were mixed on whether AI would lead to inequalities in care.

#### 3.4.2 Efficiency

Over half of HCPs agreed that AI could lead to quicker referrals to hospital eye services and improve the efficiency of the DESP. 60% believed AI could deliver vast amounts of high‐quality data in real‐time, and 70% thought AI could lead to quicker reporting of outcomes. However, less than 45% thought AI could reduce errors in retinal image grading.

#### 3.4.3 Impact on the workforce

HCPs largely agreed that AI could reduce the time spent on repetitive tasks (65%), free up time for patient care (64%), help address workforce shortages in the DESP (62%) and partly replace human grading (71%). However, HCPs expressed concerns about AI decreasing reliance on humans for screening, reducing training opportunities and negatively affecting job satisfaction. 66% of HCP respondents expressed concern about benchmarking human performance against AI, and 71% disagreed that AI could wholly replace human grading.

#### 3.4.4 Data security/responsibility

There were significant concerns among HCPs about how AI would be quality checked in the DESP, who would be responsible for errors, and the governance and regulation of AI systems (Figure 5). A majority (76%) were concerned about the regulation of AI, and 62% worried about healthcare data being used for commercial purposes.

#### 3.4.5 Adjusted differences in Likert scores by subgroups of HCP

Differences in Likert scores are summarised in Table 4. Compared with BARS, HCPs were more concerned about data security, significantly so for Tyneside (adjusted difference −0.4). Women HCPs were more concerned about efficiency, data security and the negative impacts on the workforce compared with men. Younger HCPs were less concerned about data security. HCPs from Asian backgrounds showed greater concerns across all themes, especially about data security.

**TABLE 4:** Multivariable linear regression results for healthcare professionals.

Differences in scores compared to base group (95% confidence interval)							
Characteristics	General score	Efficiency score	Data security score	Trust score	Workforce (+) score	Workforce (−) score	Screening experience
Site: Base: BARS							
North East London	0.02 (−0.27, 0.30)	0.22 (−0.10, 0.54)	−0.28 (−0.57, 0.01)	−0.04 (−0.37, 0.29)	0.08 (−0.23, 0.39)	−0.15 (−0.49, 0.18)	−0.01 (−0.31, 0.28)
South East London	−0.25 (−0.56, 0.06)	0.05 (−0.30, 0.40)	−0.12 (−0.44, 0.20)	−0.03 (−0.40, 0.33)	0.04 (−0.31, 0.38)	−0.16 (−0.53, 0.21)	0.00 (−0.33, 0.32)
Gloucester	0.14 (−0.14, 0.43)	0.24 (−0.09, 0.56)	−0.28 (−0.58, 0.02)	0.09 (−0.25, 0.43)	0.46 (0.14, 0.78)^†^	0.13 (−0.21, 0.47)	0.23 (−0.07, 0.54)
Tyneside	0.06 (−0.29, 0.41)	0.03 (−0.37, 0.43)	−0.43 (−0.80, −0.07)*	0.10 (−0.32, 0.52)	0.30 (−0.10, 0.69)	−0.23 (−0.65, 0.19)	−0.17 (−0.55, 0.20)
Age group: Base: ≥50 to <60 years							
<30 years	0.05 (−0.28, 0.38)	0.11 (−0.27, 0.48)	0.30 (−0.05, 0.64)	0.00 (−0.39, 0.39)	0.09 (−0.27, 0.46)	−0.34 (−0.74, 0.05)	−0.12 (−0.47, 0.23)
≥30 to <40 years	0.05 (−0.20, 0.30)	0.09 (−0.19, 0.38)	0.09 (−0.17, 0.35)	−0.03 (−0.33, 0.26)	−0.10 (−0.38, 0.18)	−0.17 (−0.47, 0.13)	−0.16 (−0.42, 0.10)
≥40 to <50 years	0.07 (−0.18, 0.32)	−0.05 (−0.33, 0.23)	0.35 (0.09, 0.61)*	−0.06 (−0.35, 0.24)	−0.09 (−0.37, 0.18)	0.16 (−0.14, 0.46)	0.00 (−0.27, 0.26)
60+ years	0.32 (0.01, 0.63)*	0.23 (−0.12, 0.58)	−0.02 (−0.34, 0.31)	0.29 (−0.08, 0.66)	0.11 (−0.24, 0.45)	0.14 (−0.23, 0.51)	0.11 (−0.22, 0.44)
Sex: Base: Men							
Woman	−0.17 (−0.37, 0.02)	−0.22 (−0.44, 0.00)*	−0.20 (−0.40, 0.00)*	−0.19 (−0.42, 0.04)	−0.13 (−0.34, 0.09)	−0.41 (−0.64, −0.18)^ⱡ^	−0.18 (−0.39, 0.02)
Prefer not to say	−0.46 (−1.48, 0.55)	−0.83 (−1.98, 0.32)	−0.72 (−1.77, 0.34)	−0.81 (−2.02, 0.39)	−0.31 (−1.43, 0.82)	0.26 (−0.96, 1.47)	−0.60 (−1.67, 0.47)
Ethnicity: Base: White							
Black	−0.33 (−0.90, 0.24)	−0.11 (−0.76, 0.53)	−0.21 (−0.80, 0.38)	−0.25 (−0.92, 0.43)	−0.06 (−0.69, 0.57)	0.13 (−0.55, 0.81)	0.07 (−0.53, 0.67)
Asian	−0.40 (−0.68, −0.12)^†^	−0.38 (−0.70, −0.06)*	−0.64 (−0.93, −0.35)^ⱡ^	−0.53 (−0.86, −0.20)^†^	−0.21 (−0.52, 0.10)	−0.60 (−0.93, −0.26)^ⱡ^	−0.42 (−0.71, −0.12)*
Mixed/other/prefer not to say	0.34 (−0.03, 0.71)	0.05 (−0.36, 0.47)	−0.13 (−0.51, 0.25)	0.38 (−0.05, 0.82)	0.10 (−0.31, 0.51)	−0.28 (−0.71, 0.16)	0.06 (−0.32, 0.45)
Townsend score: Base: ≥−0.5 to <2							
<−2 (least deprived)	−0.05 (−0.33, 0.22)	0.02 (−0.29, 0.33)	0.05 (−0.23, 0.33)	−0.12 (−0.44, 0.20)	0.10 (−0.20, 0.40)	−0.33 (−0.65, −0.01)*	0.00 (−0.29, 0.28)
≥−2 to <−0.5	−0.21 (−0.48, 0.06)	−0.22 (−0.53, 0.09)	−0.01 (−0.29, 0.28)	−0.36 (−0.68, −0.03)*	−0.25 (−0.55, 0.05)	−0.35 (−0.68, −0.02)*	−0.03 (−0.32, 0.26)
≥2 to <4	−0.22 (−0.52, 0.07)	−0.35 (−0.68, −0.02)*	0.04 (−0.27, 0.34)	−0.44 (−0.79, −0.10)*	−0.18 (−0.51, 0.14)	−0.28 (−0.62, 0.07)	−0.20 (−0.51, 0.10)
≥4.0 (most deprived)	0.10 (−0.20, 0.40)	−0.09 (−0.42, 0.25)	0.15 (−0.16, 0.46)	−0.11 (−0.46, 0.25)	0.21 (−0.12, 0.54)	0.06 (−0.29, 0.42)	−0.10 (−0.42, 0.21)
Missing	0.29 (−0.53, 1.11)	0.24 (−0.69, 1.16)	0.07 (−0.78, 0.92)	0.74 (−0.23, 1.71)	0.54 (−0.37, 1.44)	−0.14 (−1.12, 0.84)	0.31 (−0.56, 1.17)
Role: Base: Screener, grader, photographer, SLTBe or optometrist							
Clinical lead or management position	0.33 (0.10, 0.55)^†^	0.32 (0.06, 0.57)*	0.01 (−0.23, 0.24)	0.22 (−0.05, 0.49)	0.34 (0.09, 0.60)*	0.20 (−0.07, 0.47)	0.18 (−0.05, 0.42)
Senior screener/grader	−0.02 (−0.25, 0.21)	−0.02 (−0.28, 0.25)	0.18 (−0.06, 0.43)	−0.05 (−0.33, 0.23)	0.09 (−0.17, 0.34)	0.11 (−0.17, 0.39)	0.06 (−0.19, 0.31)
Administrator, fail safe officer, IT officer or other	0.12 (−0.17, 0.42)	−0.03 (−0.37, 0.30)	0.40 (0.09, 0.71)*	0.21 (−0.14, 0.57)	−0.06 (−0.39, 0.27)	0.30 (−0.06, 0.65)	0.23 (−0.08, 0.55)
Length in current role: Base: Less than 5 years							
5 to less than 10 years	0.00 (−0.22, 0.22)	−0.03 (−0.28, 0.22)	−0.08 (−0.32, 0.15)	−0.06 (−0.33, 0.20)	0.00 (−0.25, 0.24)	−0.20 (−0.47, 0.07)	−0.09 (−0.32, 0.15)
10 years or more	−0.36 (−0.59, −0.13)^†^	−0.46 (−0.72, −0.20)^ⱡ^	0.01 (−0.23, 0.24)	−0.51 (−0.78, −0.24)^ⱡ^	−0.28 (−0.53, −0.02)*	−0.32 (−0.59, −0.04)*	−0.33 (−0.57, −0.09)*
Technology in daily life: Base: 5 or more applications							
Less than 5 applications	−0.34 (−0.63, −0.05)*	−0.38 (−0.71, −0.05)*	0.10 (−0.20, 0.41)	−0.26 (−0.60, 0.09)	−0.42 (−0.74, −0.09)*	−0.06 (−0.40, 0.29)	−0.13 (−0.44, 0.17)
Use of health‐based apps: Base: yes							
No	0.00 (−0.18, 0.18)	−0.12 (−0.32, 0.08)	0.06 (−0.13, 0.25)	0.03 (−0.18, 0.25)	−0.10 (−0.30, 0.10)	−0.13 (−0.34, 0.09)	−0.01 (−0.20, 0.18)

* 0.05 ≤ *p*‐value ≥0.01.

^†^ 0.01 > p‐value ≥0.001.

^ⱡ^ *p* < 0.001.

Among HCP, graders tended to report more positive attitudes compared to programme managers and clinical leads. Those who had worked in the DESP for over 10 years tended to respond less positively compared with those with less experience. HCPs who used fewer applications daily also had more negative views on general, efficiency and workforce‐related themes.

HCP and PLD using multiple applications, generally had more positive views (Tables 3 and 4).

### 3.5 Standalone questions

Logistic regression analyses (Tables S3 and S4) showed that PLD aged 60+ were less likely than younger groups to prioritise receiving screening results on the same day. Women and those who used fewer than five applications daily were more likely to agree that not understanding AI was a barrier to its acceptance. Additionally, PLD from Black and Asian ethnicities were more likely to express concerns about AI and less likely to prioritise receiving results on the same day.

HCPs under 30 and over 60 were more likely to agree that AI could help identify diabetic eye disease. Sensitivity analyses on those who used health‐related apps revealed similar patterns (Tables S5–S8).

Box 2 outlines the five most important things PLD and HCPs wanted to learn about AI. For PLD, a focus on understanding the equitability and reliability of AI outcomes, ensuring privacy and hearing success stories from other programmes, and for HCPs—implementation logistics, AI safety, impact on their roles and changes to the screening process.

BOX 2 **Five things that PLD and HCP felt were most important to know about if AI were to be introduced into the DESP.**

**Table:** 

Key things PLD would like to know if AI were to be introduced into the diabetic eye screening programme	Key things HCPs would like to know if AI were to be introduced into the diabetic eye screening programme
Confidence in AI to work well across all people, ‘this is going to work for me’, ‘this is going to work for everyone’ Reliability of the data and evidence base behind decision to use AI—ensures trust Success of AI/Automated retinal image analysis systems in other countries Data security issues—assurance that private patient information (names, contact details) will not be sold to third parties (happy with data being used in a ‘data pool’ for research but not for commercial use) Where AI would be used within the screening process and what the screening process would look like—is there a second review/opinion?	◦ How would implementation affect the workforce—‘does it replace humans?’ ‘Is my role safe?’ ‘Will there be less need for me?’ not just immediately, but also as time goes on and software is improved—5‐/10‐year perspective ◦ Timeline of implementation, along with schedule for implementation including training etc. when and how will it be implemented ◦ Safety of the AI systems—accountability if there was a problem—who would be responsible, and what systems would be in place to identify this? Will there be ongoing quality assurance and protocols to monitor AI performance? ◦ Where AI would be used within the screening process and what the screening process would look like—is there a second review/opinion? Would patient screening experience change? ◦ Of the cases where retinal disease is indicated, will human graders review the image?

## 4 DISCUSSION

The co‐design process has been integral to this study. We believe these are the first surveys to explore the perceptions of PLD and HCP regarding AI use in NHS DESP.

Both PLD and HCP agreed that AI could improve the efficiency of the NHS DESP, with quicker reporting compared with a fully human‐led programme. Despite this, most PLD believed screening should remain human‐led. This aligns with the public's acceptance of AI in other medical diagnostics, including skin^32^ and breast cancer.^33^ A recent survey of over 7200 people in the United Kingdom found that most would not support AI in diagnosis unless NHS staff checked the results.^34^

Concerns, especially among Black and Asian PLD and HCP, focused on trust, data security, governance and accountability for AI errors. This mirrors findings from another study of HCPs, which highlighted the need for clear regulations around AI use.^35^ Additionally, Asian and Black ethnicities had a less positive perception of AI compared with white ethnicities. Contextual factors, such as differing healthcare systems, AI policy debates and structural inequities, may explain why ethnic subgroup differences diverge from a US study, where Asian respondents reported lower levels of distrust.^36^ This study also showed that women were generally less trusting of AI, which is consistent with our findings that both women PLD and HCP had more negative views on AI. Gender differences in trust warrant further exploration. Literature suggests women may report lower trust in digital health due to heightened risk perception, safety concerns and prior negative experiences of technology in healthcare. A recent Health foundation study found that women were less likely to believe AI would improve healthcare quality.^34^

PLD expressed concerns about the depersonalisation of the screening process, a concern raised in other studies on AI in healthcare in both PLD^32‐34,36–37^ and HCP.^34,35^ Interestingly, our study found that PLD who had lived longer with diabetes were more accepting of AI. PLD and HCP who used five or more online applications daily were generally more positive about incorporating AI into the DESP, particularly those who used health‐related apps. Daily app usage was included as a proxy for digital literacy and engagement, but may also reflect socio‐economic status. While models adjusted for deprivation and education, residual confounding is possible.

Among HCP, one in ten would not trust AI‐assisted screening results. Concerns were raised regarding job satisfaction, training and career progression. While most HCP believed AI could partially replace human grading in the DESP, only a minority thought it could fully replace humans. This is consistent with a study of paediatric ophthalmologists, where most respondents believed AI would improve the field, but fewer believed it would replace clinicians.^29^ A recent survey of HCP also identified concerns about the patient–practitioner relationship and AI's accuracy.^34^ Concerns expressed by more experienced HCP likely reflect professional identity, job security, and scepticism about workforce shifts. This suggests that AI adoption strategies should directly address role‐specific anxieties.

Most PLD were open to having their data used for research to improve health services, provided the research was controlled by the NHS. Key information needed to inform PLD and HCP about the introduction of AI into the DESP was identified (Box 2).

A major strength of this study is its large‐scale co‐designed survey across both PLD and HCP. However, limitations include a relatively low PLD response rate (4.8%), reliance on online distribution in English, and potential underrepresentation of digitally excluded groups or those with limited English literacy. Respondents may therefore represent a more motivated and technologically engaged sample, and the attitudes observed here may be more positive than in the wider PLD population.

The findings align closely with NHS AI policy priorities around safety, transparency and accountability, suggesting that respondents' concerns map directly onto ongoing governance frameworks.^38^

The findings of this study were corroborated through complementary qualitative analysis of survey free‐text responses. Within survey framing, AI was described as an assistive triage tool, although free‐text responses indicated this distinction was not always clear.^39^

Potential limitations of the surveys include selection bias and underrepresentation of people who do not speak English as a first language or those without access to the internet. Nonetheless, the data includes responses from a broad range of population subgroups. Several UK academic articles examining perceptions of AI among staff or patients either report modest‐to‐high response rates when the denominator is well defined (e.g. local paper‐based/clinic surveys) or do not report a denominator when using convenience recruitment or open web links. Examples vary from ~1% up to 70%+ depending on mode and population.^40,41^ These align with response rates from HCP (~71%) and PLD (~4.8%).

Although the surveys focused on diabetic eye screening, we included items on general technology use. However, prior exposure to generative AI was not directly assessed and may represent an important contextual factor shaping responses. In addition, the timing of the survey coincided with heightened public debate around generative AI, which may have influenced perceptions and introduced external framing effects beyond diabetic eye screening.

There is no ideal methodology for quantifying perceptions, and Likert scales may be subject to acquiescence bias. However, Likert scales are considered effective for psychometric testing^42^ and have been used in similar studies.^25^ The use of Likert scales facilitated data visualisation and analysis. Linear and logistic regression approaches were used to ensure coherence. To minimise acquiescence bias, survey items were worded both positively and negatively within the same question group.

Although we modelled Likert theme scores using linear regression, treating them as approximately continuous variables, this approach is supported by prior health survey methodology. Alternative ordinal logistic regression was considered but not used, as our primary outcomes were aggregated mean scores across multiple items, which approximate normal distributions.

## 5 CONCLUSIONS

These co‐designed surveys highlighted key areas that need to be addressed in outreach activities to support the integration of AI into the DESP. Targeted outreach strategies should include (i) community‐based engagement and education to build trust among PLD from minority ethnic groups; (ii) training and clear role delineation to address HCP concerns; and (iii) transparent communication on data use, safety, and oversight. These activities should also include education about the purpose of AI, how it will be integrated into the screening process, the level of human oversight and reassurance that AI will not reduce human contact during screening appointments.

Certain sociodemographic groups expressed specific concerns that should be addressed to avoid mistrust or disengagement. For PLD, the highest priorities are equity, reliability and privacy. For HCP, clarity on safety, accountability and workforce implications is key. Addressing these concerns will be essential for equitable and sustainable AI adoption in screening. In particular, PLD need assurances that AI will be effective across all population subgroups, that their personal data will not be sold to third parties, and that there is evidence of successful AI use in other contexts. HCP raised concerns about job security, resource allocation, benchmarking human graders against AI and the need for training and career development. A clear timeline for AI implementation, including training, and addressing safety and responsibility concerns is crucial to building trust among HCPs. Future work will focus on developing outreach educational resources to address the barriers and enablers of AI acceptance in diabetic eye screening.

## AUTHOR CONTRIBUTIONS

All authors, including members of the ARIAS research group consortium contributed to this manuscript. KW, LC, UC, CW, LB, RC, JA, CGO and ARR drafted the study design. All co‐authors contributed to iterative updates. KW, RS and ARR wrote the first draft of the report, which all co‐authors contributed to and critically appraised. ARR and CGO are responsible for data integrity and will act as guarantors.

*The Artificial Intelligence/Automated Retinal Image Analysis Systems (ARIAS) Research Group*: John Anderson, Sarah Barman, Louis Bolter, Tom Broad, Ryan Chambers, Lakshmi Chandrasekaran, Umar Chaudhry, Miu Chi Tang, Clare Connor, Karen Easy, Cathy Egan, Davies Eju‐Konem, Jiri Fajtl, Maged Habib, Julie Hapeshi, Aaron Lee, Laura Lodge, Fiona Martin, Samantha Mann, Peter Mitchell, Abdul Mulla Gbenga Olasehinde, Abraham Olvera‐Barrios, Christopher G Owen, Ahmed Patel, Alicja R Rudnicka, Peter Scanlon, Adam Stott, Adnan Tufail, Charlotte Wahlich, Laura Webster, Roshan Welikala and Kathryn Willis.

## FUNDING INFORMATION

This work was funded by NHS Transformation Directorate and The Health Foundation (and managed by the National Institute for Health and Social Care Research: AI_HI200008). The views expressed in this publication are those of the author(s) and not necessarily those of the NHS Transformation Directorate, The Health Foundation, National Institute for Health Research or the Department of Health and Social Care. Other funders included the St Georges, University of London Participatory Fund and a Wellcome Collaborative Award in Science (2022, 224390/Z/21/Z). LC is funded by an NIHR In‐Practice Fellowship (NIHR305176).

## CONFLICT OF INTEREST STATEMENT

None of the authors have any competing interests.

## Supporting information


**Figure S1.** Response rates for people living with diabetes and healthcare practitioners by centre.
**Table S1**. People living with diabetes survey questions and responses by site.
**Table S2**. Healthcare practitioner survey questions and responses by site.
**Table S3**. Multivariable logistic regression results for people living with diabetes.
**Table S4**. Multivariable logistic regression results for health care practitioners.
**Table S5**. Multivariable linear regression results among health app users (people living with diabetes).
**Table S6**. Multivariable logistic regression results among health app users (people living with diabetes).
**Table S7**. Multivariable linear regression results among health app users (health care practitioners).
**Table S8**. Multivariable logistic regression results among health app users (health care practitioners).
**Text S1**. Sample size.
**Text S2**. Additional references.
