# Supplementary material for: What are the perceptions and concerns of people living with diabetes and National Health Service staff around the potential implementation of AI‐assisted screening for diabetic eye disease?
Source: Diabet Med. 2025 Nov 10;43(1):e70165. doi: 10.1111/dme.70165 (PMC12700512; doi:10.1111/dme.70165)
Supplement: Supplementary file 1 — Figure S1. Response rates for people living with diabetes and healthcare practitioners by centre. Table S1. People living with diabetes survey questions and responses by site. Table S2. Healthcare practitioner survey questions and responses by site. Table S3. Multivariable logistic regression results for people living with diabetes. Table S4. Multivariable logistic regression results for health care practitioners. Table S5. Multivariable linear regression results among health app users (people living with diabetes). Table S6. Multivariable logistic regression results among health app users (people living with diabetes). Table S7. Multivariable linear regression results among health app users (health care practitioners). Table S8. Multivariable logistic regression results among health app users (health care practitioners). Text S1. Sample size. Text S2. Additional references. [file DME-43-e70165-s001.docx]

**SUPPLEMENTARY MATERIAL**

# What are the perceptions and concerns of people living with diabetes and National Health Service staff around the potential implementation of AI-assisted screening for diabetic eye disease? Quantitative results of two cross-sectional surveys in a secondary care screening setting

Kathryn Willis†, Royce Shakespeare†, Lakshmi Chandrasekaran†, Umar Chaudhry†, Charlotte Wahlich†, Ryan Chambers*, Louis Bolter*, John Anderson*, Abraham Olvera-Barrios‡, Jiri Fajtl§, Roshan Welikala§, Sarah Barman§, Samantha Mann††, Peter Scanlon‡‡, Maged S Habib#, Catherine A Egan‡, Adnan Tufail‡, Christopher G Owen†, Alicja R Rudnicka†

*On behalf of the ARIAS Research Group*

† Population Health Research Institute, St George’s School of Health and Medical Sciences, City St George’s, University of London, London, UK

*Homerton Healthcare NHS Foundation Trust, London, UK

‡ NIHR Biomedical Research Centre, Moorfields Eye Hospital NHS Foundation Trust and Institute of Ophthalmology University College London, London, UK

§ School of Computer Science and Mathematics, Kingston University, London, UK

†† Guy’s and St Thomas’s NHS Foundation Trust

‡‡ Gloucestershire Hospitals NHS Foundation Trust

# South Tyneside and Sunderland NHS Foundation Trust

Contents[_Toc198562402](#_Toc198562402)

[Figure S1: Response rates for people living with diabetes and Healthcare practitioners by centre. 2](#_Toc198562403)

[Text S1: Sample Size 2](#_Toc198562404)

[Table S1: People living with diabetes survey questions and responses by site 3](#_Toc198562405)

[Table S2 Healthcare Practitioner survey questions and responses by site 5](#_Toc198562406)

[Table S3: Multivariable logistic regression results for people living with diabetes 8](#_Toc198562407)

[Table S4: Multivariable logistic regression results for health care practitioners 11](#_Toc198562408)

[Table S5: Multivariable Linear regression results among health app users (people living with diabetes) 13](#_Toc198562409)

[Table S6: Multivariable logistic regression results among health app users (people living with diabetes) 15](#_Toc198562410)

[Table S7: Multivariable Linear regression results among health app users (health care practitioners) 17](#_Toc198562411)

[Table S8: Multivariable Logistic regression results among health app users (health care practitioners) 19](#_Toc198562412)

[Text S2: Additional References 21](#_Toc198562413)

South East London DESP

**PLD survey**

Text message invites (n): 13,920

Responses (n): 972

Response rate: 7.0%

95% CI: 6.6%-7.4%

North East London DESP

**PLD survey**

Text message invites (n): 18,163

Responses (n): 560

Response rate: 3.1%

95% CI: 2.8%-3.3%

Gloucestershire

DESP

South Tyneside

DESP

**PLD survey**

*Not distributed*

**HCP survey**

Staff invited (n): 35

Responses (n): 30

Response rate: 85.7%

95% CI: 69.7%-95.2%

**PLD survey**

Leaflet invites (n): 1000

Responses (n): 35

Response rate: 3.5%

95% CI: 2.4%-4.8%

**HCP survey**

Staff invited (n): 78

Responses (n): 55

Response rate: 70.5%

95% CI: 60.5%-81.5%

**HCP survey**

Staff invited (n): 54

Responses (n): 30

Response rate: 55.6%

95% CI: 41.4%-69.1%

**HCP survey**

Staff invited (n): 25

Responses (n): 22

Response rate: 88.0%

95% CI: 68.8%-97.5%

## Figure S1: Response rates for people living with diabetes and Healthcare practitioners by centre.

## Text S1: Sample Size

We aimed to recruit 100 PLD from each of the 3 main ethnic sub-groups to detect a one-step difference in mean Likert scores between groups (assuming a common standard deviation of 1.5) with 95% power and alpha (type 1 error) set to 0.01.[30] Each sociodemographic factor, including age, sex and Townsend scores as a measure of deprivation[32], was grouped into a maximum of 5 subgroups for analysis, thereby providing approximately 60 participants per subgroup. This reduced the power to 90-95% (dependent on sub-group) and alpha to 0.05 to detect one-step difference in mean Likert scores between sub-groups.

Based on previous survey work, we anticipated a 1% response rate to the surveys from PLD and hence three large DESPs were used as recruitment sites with experience in survey distribution within their screening programme. For HCP, we did not stipulate target sample sizes for staff by sociodemographic groups but aimed to recruit 70-80% of staff across NHS DESPs in this study.

## Table S1: People living with diabetes survey questions and responses by site

| **Stand Alone Questions** | | | **Total** | **NEL** | **SEL** | **Gloucester** | **Other** |
| --- | --- | --- | --- | --- | --- | --- | --- |
|  |  |  | n (%) | n (%) | n (%) | n (%) | n (%) |
| Q5 | ''The Diabetic Eye Screening Programme is important in monitoring my condition.' | Disagree or strongly disagree | 4 (0.3) | 2 (0.4) | 2 (0.2) | 0 (0.0) | 0 (0.0) |
|  |  | Neither agree nor disagree | 20 (1.3) | 9 (1.6) | 11 (1.1) | 0 (0.0) | 0 (0.0) |
|  |  | Agree or strongly agree | 1,548 (98.5) | 547 (98.0) | 954 (98.7) | 35 (100.0) | 12 (100.0) |
| Q17 | “'I am concerned that not knowing how AI works is a barrier to people living with diabetes accepting this technology for diabetic eye screening” | Disagree or strongly disagree | 186 (11.8) | 59 (10.6) | 120 (12.3) | 4 (11.4) | 3 (25.0) |
|  |  | Neither agree nor disagree | 459 (29.1) | 152 (27.2) | 292 (30.0) | 13 (37.1) | 2 (16.7) |
|  |  | Agree or strongly agree | 932 (59.1) | 347 (62.2) | 560 (57.6) | 18 (51.4) | 7 (58.3) |
| Q21 | “If my eye images were processed by a computer or AI technology, I would be happy for these data to be securely stored under the control of the NHS and used for research to do with healthcare evaluation or improvement” | Disagree or strongly disagree | 93 (5.9) | 34 (6.1) | 56 (5.8) | 2 (5.7) | 1 (8.3) |
|  |  | Neither agree nor disagree | 253 (16.0) | 89 (15.9) | 160 (16.5) | 3 (8.6) | 1 (8.3) |
|  |  | Agree or strongly agree | 1,231 (78.1) | 435 (78.0) | 756 (77.8) | 30 (85.7) | 10 (83.3) |
| Q23 | “I believe AI could detect diabetic eye disease equally well in people of different ethnic groups and different ages” | Disagree or strongly disagree | 95 (6.0) | 25 (4.5) | 66 (6.8) | 3 (8.6) | 1 (8.3) |
|  |  | Neither agree nor disagree | 574 (36.4) | 187 (33.5) | 373 (38.4) | 9 (25.7) | 5 (41.7) |
|  |  | Agree or strongly agree | 908 (57.6) | 346 (62.0) | 533 (54.8) | 23 (65.7) | 6 (50.0) |
| Q24_3_a | “It would be important for me to receive the results of my eye assessment on the day” | Disagree or strongly disagree | 205 (13.0) | 59 (10.6) | 139 (14.3) | 4 (11.4) | 3 (25.0) |
|  |  | Neither agree nor disagree | 587 (37.2) | 192 (34.4) | 372 (38.3) | 18 (51.4) | 5 (41.7) |
|  |  | Agree or strongly agree | 785 (49.8) | 307 (55.0) | 461 (47.4) | 13 (37.1) | 4 (33.3) |
| Q24_4_a | “I would be happy to receive results from my eye assessment via a text message or email rather than in a letter” | Disagree or strongly disagree | 169 (10.7) | 48 (8.6) | 120 (12.3) | 1 (2.9) | 0 (0.0) |
|  |  | Neither agree nor disagree | 310 (19.7) | 110 (19.7) | 196 (20.2) | 4 (11.4) | 0 (0.0) |
|  |  | Agree or strongly agree | 1,098 (69.6) | 400 (71.7) | 656 (67.5) | 30 (85.7) | 12 (100.0) |
|  | **General Questions** | |  |  |  |  |  |
| Q15 | “I believe AI could help identify diabetic eye disease” | Disagree or strongly disagree | 61 (3.9) | 27 (4.8) | 33 (3.4) | 1 (2.9) | 0 (0.0) |
|  |  | Neither agree nor disagree | 423 (26.8) | 144 (25.8) | 270 (27.8) | 7 (20.0) | 2 (16.7) |
|  |  | Agree or strongly agree | 1,093 (69.3) | 387 (69.4) | 669 (68.8) | 27 (77.1) | 10 (83.3) |
| Q16_1_a | “I believe the use of AI in diabetic eye screening could...”:  …save money for the NHS | Disagree or strongly disagree | 75 (4.8) | 32 (5.7) | 42 (4.3) | 1 (2.9) | 0 (0.0) |
|  |  | Neither agree nor disagree | 435 (27.6) | 142 (25.4) | 283 (29.1) | 8 (22.9) | 2 (16.7) |
|  |  | Agree or strongly agree | 1,067 (67.7) | 384 (68.8) | 647 (66.6) | 26 (74.3) | 10 (83.3) |
| Q16_2_a | “I believe the use of AI in diabetic eye screening could...”:  …improve accuracy and reliability of screening | Disagree or strongly disagree | 95 (6.0) | 36 (6.5) | 55 (5.7) | 2 (5.7) | 2 (16.7) |
|  |  | Neither agree nor disagree | 463 (29.4) | 149 (26.7) | 304 (31.3) | 8 (22.9) | 2 (16.7) |
|  |  | Agree or strongly agree | 1,019 (64.6) | 373 (66.8) | 613 (63.1) | 25 (71.4) | 8 (66.7) |
| Q16_3_a | “I believe the use of AI in diabetic eye screening could...”:  …decrease reliance on healthcare practitioners screening for diabetic eye disease | Disagree or strongly disagree | 147 (9.3) | 57 (10.2) | 88 (9.1) | 2 (5.7) | 0 (0.0) |
|  |  | Neither agree nor disagree | 473 (30.0) | 141 (25.3) | 316 (32.5) | 11 (31.4) | 5 (41.7) |
|  |  | Agree or strongly agree | 957 (60.7) | 360 (64.5) | 568 (58.4) | 22 (62.9) | 7 (58.3) |
| **Efficiency** | | |  |  |  |  |  |
| Q19_1_a | I think the use of AI could lead to quicker results from the eye assessment | Disagree or strongly disagree | 61 (3.9) | 22 (3.9) | 38 (3.9) | 1 (2.9) | 0 (0.0) |
|  |  | Neither agree nor disagree | 439 (27.8) | 148 (26.5) | 277 (28.5) | 9 (25.7) | 5 (41.7) |
|  |  | Agree or strongly agree | 1,077 (68.3) | 388 (69.5) | 657 (67.6) | 25 (71.4) | 7 (58.3) |
| Q19_2_a | I believe the use of AI could free up time for staff to work on other areas of patient care | Disagree or strongly disagree | 109 (6.9) | 35 (6.3) | 71 (7.3) | 3 (8.6) | 0 (0.0) |
|  |  | Neither agree nor disagree | 394 (25.0) | 145 (26.0) | 244 (25.1) | 4 (11.4) | 1 (8.3) |
|  |  | Agree or strongly agree | 1,074 (68.1) | 378 (67.7) | 657 (67.6) | 28 (80.0) | 11 (91.7) |
| Q19_3_a | I would prefer screening that is human-led, which could mean a longer waiting time for results | Disagree or strongly disagree | 319 (20.2) | 89 (15.9) | 210 (21.6) | 14 (40.0) | 6 (50.0) |
|  |  | Neither agree nor disagree | 562 (35.6) | 175 (31.4) | 372 (38.3) | 11 (31.4) | 4 (33.3) |
|  |  | Agree or strongly agree | 696 (44.1) | 294 (52.7) | 390 (40.1) | 10 (28.6) | 2 (16.7) |
| **Data Security** | | |  |  |  |  |  |
| Q20_1_a | “If AI were to be introduced into the diabetic eye screening programme, I would be concerned about…”:  ...the confidentiality of my personal information | Disagree or strongly disagree | 333 (21.1) | 94 (16.8) | 222 (22.8) | 10 (28.6) | 7 (58.3) |
|  |  | Neither agree nor disagree | 511 (32.4) | 157 (28.1) | 336 (34.6) | 16 (45.7) | 2 (16.7) |
|  |  | Agree or strongly agree | 733 (46.5) | 307 (55.0) | 414 (42.6) | 9 (25.7) | 3 (25.0) |
| Q20_2_a | “If AI were to be introduced into the diabetic eye screening programme, I would be concerned about…”:  **…**who is responsible if errors occur from the use of AI technology | Disagree or strongly disagree | 106 (6.7) | 35 (6.3) | 66 (6.8) | 4 (11.4) | 1 (8.3) |
|  |  | Neither agree nor disagree | 351 (22.3) | 120 (21.5) | 217 (22.3) | 11 (31.4) | 3 (25.0) |
|  |  | Agree or strongly agree | 1,120 (71.0) | 403 (72.2) | 689 (70.9) | 20 (57.1) | 8 (66.7) |
| Q20_3_a | “If AI were to be introduced into the diabetic eye screening programme, I would be concerned about…”:  ...how AI technology will be quality checked in the diabetic eye screening programme | Disagree or strongly disagree | 103 (6.5) | 37 (6.6) | 59 (6.1) | 4 (11.4) | 3 (25.0) |
|  |  | Neither agree nor disagree | 339 (21.5) | 97 (17.4) | 231 (23.8) | 10 (28.6) | 1 (8.3) |
|  |  | Agree or strongly agree | 1,135 (72.0) | 424 (76.0) | 682 (70.2) | 21 (60.0) | 8 (66.7) |
| **Trust** | | |  |  |  |  |  |
| Q22_1_a | I would trust the results from an AI-assisted eye assessment | Disagree or strongly disagree | 143 (9.1) | 55 (9.9) | 84 (8.6) | 2 (5.7) | 2 (16.7) |
|  |  | Neither agree nor disagree | 551 (34.9) | 180 (32.3) | 359 (36.9) | 11 (31.4) | 1 (8.3) |
|  |  | Agree or strongly agree | 883 (56.0) | 323 (57.9) | 529 (54.4) | 22 (62.9) | 9 (75.0) |
| Q22_2_a | If AI were to be introduced, humans should remain responsible for the final screening outcome | Disagree or strongly disagree | 47 (3.0) | 21 (3.8) | 25 (2.6) | 1 (2.9) | 0 (0.0) |
|  |  | Neither agree nor disagree | 255 (16.2) | 83 (14.9) | 164 (16.9) | 2 (5.7) | 6 (50.0) |
|  |  | Agree or strongly agree | 1,275 (80.8) | 454 (81.4) | 783 (80.6) | 32 (91.4) | 6 (50.0) |
| **Screening experience** | | |  |  |  |  |  |
| Q24_1_a | I am concerned that the use of AI might negatively affect the relationship between patients and staff | Disagree or strongly disagree | 367 (23.3) | 108 (19.4) | 243 (25.0) | 12 (34.3) | 4 (33.3) |
|  |  | Neither agree nor disagree | 603 (38.2) | 194 (34.8) | 386 (39.7) | 18 (51.4) | 5 (41.7) |
|  |  | Agree or strongly agree | 607 (38.5) | 256 (45.9) | 343 (35.3) | 5 (14.3) | 3 (25.0) |
| Q24_2_a | I would be comfortable with the use of AI as part of my eye assessment | Disagree or strongly disagree | 137 (8.7) | 46 (8.2) | 89 (9.2) | 1 (2.9) | 1 (8.3) |
|  |  | Neither agree nor disagree | 389 (24.7) | 139 (24.9) | 246 (25.3) | 3 (8.6) | 1 (8.3) |
|  |  | Agree or strongly agree | 1,051 (66.6) | 373 (66.8) | 637 (65.5) | 31 (88.6) | 10 (83.3) |
| Q24_5_a | I believe that using AI as part of my assessment could lead to a less personalised patient experience | Disagree or strongly disagree | 177 (11.2) | 55 (9.9) | 111 (11.4) | 6 (17.1) | 5 (41.7) |
|  |  | Neither agree nor disagree | 505 (32.0) | 155 (27.8) | 334 (34.4) | 13 (37.1) | 3 (25.0) |
|  |  | Agree or strongly agree | 895 (56.8) | 348 (62.4) | 527 (54.2) | 16 (45.7) | 4 (33.3) |

## Table S2 Healthcare Practitioner survey questions and responses by site

| **Stand Alone Questions** | | | **Total** | **NEL** | **SEL** | **Gloucester** | **S Tyne** | **BARS** |
| --- | --- | --- | --- | --- | --- | --- | --- | --- |
|  |  |  | **n (%)** | **n (%)** | **n (%)** | **n (%)** | **n (%)** | **n (%)** |
| Q11 | “A lack of transparency in how AI works is a barrier to health care professionals accepting this technology for use within the DESP” | Disagree or strongly disagree | 17 (6.5) | 3 (5.5) | 1 (3.3) | 2 (6.7) | 0 (0.0) | 11 (8.8) |
|  |  | Neither agree nor disagree | 84 (32.1) | 24 (43.6) | 9 (30.0) | 9 (30.0) | 9 (40.9) | 33 (26.4) |
|  |  | Agree or strongly agree | 161 (61.5) | 28 (50.9) | 20 (66.7) | 19 (63.3) | 13 (59.1) | 81 (64.8) |
| Q15 | “I believe AI could detect diabetic eye disease equally well in people of different ethnic groups and different ages” | Disagree or strongly disagree | 80 (30.5) | 18 (32.7) | 7 (23.3) | 11 (36.7) | 3 (13.6) | 41 (32.8) |
|  |  | Neither agree nor disagree | 99 (37.8) | 24 (43.6) | 9 (30.0) | 14 (46.7) | 10 (45.5) | 42 (33.6) |
|  |  | Agree or strongly agree | 83 (31.7) | 13 (23.6) | 14 (46.7) | 5 (16.7) | 9 (40.9) | 42 (33.6) |
| Q16_5­_a | ...wholly replace human grading of retinal images for detection of diabetic eye disease within the DESP | Disagree or strongly disagree | 185 (70.6) | 38 (69.1) | 19 (63.3) | 23 (76.7) | 11 (50.0) | 94 (75.2) |
|  |  | Neither agree nor disagree | 34 (13.0) | 9 (16.4) | 4 (13.3) | 3 (10.0) | 5 (22.7) | 13 (10.4) |
|  |  | Agree or strongly agree | 43 (16.4) | 8 (14.5) | 7 (23.3) | 4 (13.3) | 6 (27.3) | 18 (14.4) |
| Q18 | “Further training would be required for staff working in the DESP if AI systems for analysing retinal images were to be implemented” | Disagree or strongly disagree | 21 (8.0) | 2 (3.6) | 5 (16.7) | 3 (10.0) | 1 (4.5) | 10 (8.0) |
|  |  | Neither agree nor disagree | 50 (19.1) | 17 (30.9) | 7 (23.3) | 3 (10.0) | 1 (4.5) | 22 (17.6) |
|  |  | Agree or strongly agree | 191 (72.9) | 36 (65.5) | 18 (60.0) | 24 (80.0) | 20 (90.9) | 93 (74.4) |
| **General** | | |  |  |  |  |  |  |
| Q10_1_a | ...save the NHS DESP money in the long-term | Disagree or strongly disagree | 24 (9.2) | 7 (12.7) | 1 (3.3) | 0 (0.0) | 2 (9.1) | 14 (11.2) |
|  |  | Neither agree nor disagree | 63 (24.0) | 17 (30.9) | 7 (23.3) | 3 (10.0) | 1 (4.5) | 22 (17.6) |
|  |  | Agree or strongly agree | 175 (66.8) | 37 (67.3) | 18 (60.0) | 22 (73.3) | 15 (68.2) | 83 (66.4) |
| Q10_2_a | ...provide more cost-effective NHS care for those with diabetes | Disagree or strongly disagree | 34 (13.0) | 11 (20.0) | 2 (6.7) | 1 (3.3) | 3 (13.6) | 17 (13.6) |
|  |  | Neither agree nor disagree | 65 (24.8) | 12 (21.8) | 11 (36.7) | 4 (13.3) | 5 (22.7) | 33 (26.4) |
|  |  | Agree or strongly agree | 163 (62.2) | 32 (58.2) | 17 (56.7) | 25 (83.3) | 14 (63.6) | 75 (60.0) |
| Q10_3_a | ...lead to inequalities in care | Disagree or strongly disagree | 82 (31.3) | 17 (30.9) | 7 (23.3) | 11 (36.7) | 4 (18.2) | 43 (34.4) |
|  |  | Neither agree nor disagree | 89 (34.0) | 18 (32.7) | 12 (40.0) | 10 (33.3) | 8 (36.4) | 41 (32.8) |
|  |  | Agree or strongly agree | 91 (34.7) | 20 (36.4) | 11 (36.7) | 9 (30.0) | 10 (45.5) | 41 (32.8) |
| Q10_4_a | ...lead to greater use of evidence-based disease management for diabetic eye screening | Disagree or strongly disagree | 42 (16.0) | 8 (14.5) | 8 (26.7) | 4 (13.3) | 2 (9.1) | 20 (16.0) |
|  |  | Neither agree nor disagree | 98 (37.4) | 25 (45.5) | 12 (40.0) | 14 (46.7) | 8 (36.4) | 39 (31.2) |
|  |  | Agree or strongly agree | 122 (46.6) | 22 (40.0) | 10 (33.3) | 12 (40.0) | 12 (54.5) | 66 (52.8) |
| Q10_5_a | …lead to greater uniformity in screening outcomes and management decisions | Disagree or strongly disagree | 48 (18.3) | 12 (21.8) | 9 (30.0) | 6 (20.0) | 2 (9.1) | 19 (15.2) |
|  |  | Neither agree nor disagree | 82 (31.3) | 19 (34.5) | 12 (40.0) | 11 (36.7) | 7 (31.8) | 33 (26.4) |
|  |  | Agree or strongly agree | 132 (50.4) | 24 (43.6) | 9 (30.0) | 13 (43.3) | 13 (59.1) | 73 (58.4) |
| Q10_6_a | ...assist with the grading of diabetic retinopathy within the DESP | Disagree or strongly disagree | 28 (10.7) | 7 (12.7) | 3 (10.0) | 0 (0.0) | 2 (9.1) | 16 (12.8) |
|  |  | Neither agree nor disagree | 40 (15.3) | 11 (20.0) | 6 (20.0) | 3 (10.0) | 6 (27.3) | 14 (11.2) |
|  |  | Agree or strongly agree | 194 (74.0) | 37 (67.3) | 21 (70.0) | 27 (90.0) | 14 (63.6) | 95 (76.0) |
| **Efficiency** | | |  |  |  |  |  |  |
| Q12_1_a | ...help reduce errors in grading of retinal images | Disagree or strongly disagree | 73 (27.9) | 16 (29.1) | 9 (30.0) | 9 (30.0) | 6 (27.3) | 33 (26.4) |
|  |  | Neither agree nor disagree | 75 (28.6) | 20 (36.4) | 7 (23.3) | 9 (30.0) | 5 (22.7) | 34 (27.2) |
|  |  | Agree or strongly agree | 114 (43.5) | 19 (34.5) | 14 (46.7) | 12 (40.0) | 11 (50.0) | 58 (46.4) |
| Q12_2_a | ...rapidly deliver vast amounts of clinically relevant, high-quality data in real time | Disagree or strongly disagree | 26 (9.9) | 6 (10.9) | 3 (10.0) | 1 (3.3) | 3 (13.6) | 13 (10.4) |
|  |  | Neither agree nor disagree | 72 (27.5) | 19 (34.5) | 7 (23.3) | 9 (30.0) | 8 (36.4) | 29 (23.2) |
|  |  | Agree or strongly agree | 164 (62.6) | 30 (54.5) | 20 (66.7) | 20 (66.7) | 11 (50.0) | 83 (66.4) |
| Q12_3_a | ...make the DESP run more efficiently | Disagree or strongly disagree | 43 (16.4) | 10 (18.2) | 4 (13.3) | 2 (6.7) | 4 (18.2) | 23 (18.4) |
|  |  | Neither agree nor disagree | 80 (30.5) | 21 (38.2) | 8 (26.7) | 9 (30.0) | 8 (36.4) | 34 (27.2) |
|  |  | Agree or strongly agree | 139 (53.1) | 24 (43.6) | 18 (60.0) | 19 (63.3) | 10 (45.5) | 68 (54.4) |
| Q12_4_a | ...lead to quicker reporting of screening outcomes | Disagree or strongly disagree | 32 (12.2) | 4 (7.3) | 4 (13.3) | 1 (3.3) | 5 (22.7) | 18 (14.4) |
|  |  | Neither agree nor disagree | 48 (18.3) | 12 (21.8) | 5 (16.7) | 3 (10.0) | 7 (31.8) | 21 (16.8) |
|  |  | Agree or strongly agree | 182 (69.5) | 39 (70.9) | 21 (70.0) | 26 (86.7) | 10 (45.5) | 86 (68.8) |
| Q12_5_a | ...lead to quicker referrals to the hospital eye service | Disagree or strongly disagree | 60 (22.9) | 8 (14.5) | 9 (30.0) | 6 (20.0) | 6 (27.3) | 31 (24.8) |
|  |  | Neither agree nor disagree | 63 (24.0) | 17 (30.9) | 6 (20.0) | 8 (26.7) | 5 (22.7) | 27 (21.6) |
|  |  | Agree or strongly agree | 139 (53.1) | 30 (54.5) | 15 (50.0) | 16 (53.3) | 11 (50.0) | 67 (53.6) |
| **Data Security** | | |  |  |  |  |  |  |
| Q13_1_a | ...the use of health care data for commercial gain | Disagree or strongly disagree | 43 (16.4) | 4 (7.3) | 4 (13.3) | 3 (10.0) | 3 (13.6) | 29 (23.2) |
|  |  | Neither agree nor disagree | 57 (21.8) | 11 (20.0) | 6 (20.0) | 5 (16.7) | 4 (18.2) | 31 (24.8) |
|  |  | Agree or strongly agree | 162 (61.8) | 40 (72.7) | 20 (66.7) | 22 (73.3) | 15 (68.2) | 65 (52.0) |
| Q13_2_a | ...patient data security and privacy | Disagree or strongly disagree | 64 (24.4) | 9 (16.4) | 7 (23.3) | 5 (16.7) | 3 (13.6) | 40 (32.0) |
|  |  | Neither agree nor disagree | 51 (19.5) | 8 (14.5) | 6 (20.0) | 8 (26.7) | 3 (13.6) | 26 (20.8) |
|  |  | Agree or strongly agree | 147 (56.1) | 38 (69.1) | 17 (56.7) | 17 (56.7) | 16 (72.7) | 59 (47.2) |
| Q13_3_a | ...regulation and governance of AI systems | Disagree or strongly disagree | 25 (9.5) | 3 (5.5) | 5 (16.7) | 1 (3.3) | 0 (0.0) | 16 (12.8) |
|  |  | Neither agree nor disagree | 39 (14.9) | 7 (12.7) | 2 (6.7) | 2 (6.7) | 3 (13.6) | 25 (20.0) |
|  |  | Agree or strongly agree | 198 (75.6) | 45 (81.8) | 23 (76.7) | 27 (90.0) | 19 (86.4) | 84 (67.2) |
| Q13_4_a | ...who is responsible if errors result from the use of AI systems | Disagree or strongly disagree | 9 (3.4) | 0 (0.0) | 2 (6.7) | 0 (0.0) | 1 (4.5) | 6 (4.8) |
|  |  | Neither agree nor disagree | 21 (8.0) | 4 (7.3) | 2 (6.7) | 2 (6.7) | 1 (4.5) | 12 (9.6) |
|  |  | Agree or strongly agree | 232 (88.5) | 51 (92.7) | 26 (86.7) | 28 (93.3) | 20 (90.9) | 107 (85.6) |
| Q13_5_a | ...how AI systems will be quality checked in the NHS DESP | Disagree or strongly disagree | 19 (7.3) | 1 (1.8) | 3 (10.0) | 0 (0.0) | 2 (9.1) | 13 (10.4) |
|  |  | Neither agree nor disagree | 20 (7.6) | 5 (9.1) | 3 (10.0) | 3 (10.0) | 2 (9.1) | 7 (5.6) |
|  |  | Agree or strongly agree | 223 (85.1) | 49 (89.1) | 24 (80.0) | 27 (90.0) | 18 (81.8) | 105 (84.0) |
| **Trust** | | |  |  |  |  |  |  |
| Q14_1_a | ...improve patient safety | Disagree or strongly disagree | 82 (31.3) | 23 (41.8) | 8 (26.7) | 7 (23.3) | 7 (31.8) | 37 (29.6) |
|  |  | Neither agree nor disagree | 104 (39.7) | 21 (38.2) | 9 (30.0) | 18 (60.0) | 8 (36.4) | 48 (38.4) |
|  |  | Agree or strongly agree | 76 (29.0) | 11 (20.0) | 13 (43.3) | 5 (16.7) | 7 (31.8) | 40 (32.0) |
| Q14_2_a | ...improve confidence in screening outcomes | Disagree or strongly disagree | 97 (37.0) | 27 (49.1) | 12 (40.0) | 9 (30.0) | 7 (31.8) | 42 (33.6) |
|  |  | Neither agree nor disagree | 100 (38.2) | 17 (30.9) | 11 (36.7) | 14 (46.7) | 8 (36.4) | 50 (40.0) |
|  |  | Agree or strongly agree | 65 (24.8) | 11 (20.0) | 7 (23.3) | 7 (23.3) | 7 (31.8) | 33 (26.4) |
| Q14_3_a | ...improve reliability of screening outcomes | Disagree or strongly disagree | 78 (29.8) | 21 (38.2) | 11 (36.7) | 8 (26.7) | 5 (22.7) | 33 (26.4) |
|  |  | Neither agree nor disagree | 89 (34.0) | 17 (30.9) | 9 (30.0) | 11 (36.7) | 8 (36.4) | 44 (35.2) |
|  |  | Agree or strongly agree | 95 (36.3) | 17 (30.9) | 10 (33.3) | 11 (36.7) | 9 (40.9) | 48 (38.4) |
| Q14_4_a | ...be as good as the current system based on human graders for the detection of diabetic retinopathy from retinal images | Disagree or strongly disagree | 84 (32.1) | 24 (43.6) | 11 (36.7) | 4 (13.3) | 5 (22.7) | 40 (32.0) |
|  |  | Neither agree nor disagree | 98 (37.4) | 16 (29.1) | 10 (33.3) | 18 (60.0) | 9 (40.9) | 45 (36.0) |
|  |  | Agree or strongly agree | 80 (30.5) | 15 (27.3) | 9 (30.0) | 8 (26.7) | 8 (36.4) | 40 (32.0) |
| **Impact on the Workforce (positively framed questions)** | | |  |  |  |  |  |  |
| Q16_1_a | ...free up time for staff to work on other areas of patient care | Disagree or strongly disagree | 35 (13.4) | 5 (9.1) | 3 (10.0) | 3 (10.0) | 3 (13.6) | 21 (16.8) |
|  |  | Neither agree nor disagree | 60 (22.9) | 18 (32.7) | 8 (26.7) | 2 (6.7) | 7 (31.8) | 25 (20.0) |
|  |  | Agree or strongly agree | 167 (63.7) | 32 (58.2) | 19 (63.3) | 25 (83.3) | 12 (54.5) | 79 (63.2) |
| Q16_2_a | ...address a workforce shortage within DESP | Disagree or strongly disagree | 42 (16.0) | 7 (12.7) | 5 (16.7) | 3 (10.0) | 3 (13.6) | 24 (19.2) |
|  |  | Neither agree nor disagree | 59 (22.5) | 12 (21.8) | 8 (26.7) | 6 (20.0) | 7 (31.8) | 26 (20.8) |
|  |  | Agree or strongly agree | 161 (61.5) | 36 (65.5) | 17 (56.7) | 21 (70.0) | 12 (54.5) | 75 (60.0) |
| Q16_3_a | ...reduce time spent by graders on repetitive screening tasks | Disagree or strongly disagree | 47 (17.9) | 10 (18.2) | 4 (13.3) | 2 (6.7) | 4 (18.2) | 27 (21.6) |
|  |  | Neither agree nor disagree | 44 (16.8) | 10 (18.2) | 9 (30.0) | 3 (10.0) | 4 (18.2) | 18 (14.4) |
|  |  | Agree or strongly agree | 171 (65.3) | 35 (63.6) | 17 (56.7) | 25 (83.3) | 14 (63.6) | 80 (64.0) |
| Q16_4_a | …partially replace human grading of retinal images for detection of diabetic eye disease within the DESP | Disagree or strongly disagree | 31 (11.8) | 9 (16.4) | 2 (6.7) | 0 (0.0) | 1 (4.5) | 19 (15.2) |
|  |  | Neither agree nor disagree | 46 (17.6) | 9 (16.4) | 9 (30.0) | 4 (13.3) | 7 (31.8) | 17 (13.6) |
|  |  | Agree or strongly agree | 185 (70.6) | 37 (67.3) | 19 (63.3) | 26 (86.7) | 14 (63.6) | 89 (71.2) |
| **Impact on the Workforce (negatively framed questions)** | | |  |  |  |  |  |  |
| Q17_1_a | ...decreasing reliance on humans for screening for diabetic retinopathy | Disagree or strongly disagree | 40 (15.3) | 3 (5.5) | 7 (23.3) | 3 (10.0) | 3 (13.6) | 24 (19.2) |
|  |  | Neither agree nor disagree | 30 (11.5) | 4 (7.3) | 3 (10.0) | 5 (16.7) | 2 (9.1) | 16 (12.8) |
|  |  | Agree or strongly agree | 192 (73.3) | 48 (87.3) | 20 (66.7) | 22 (73.3) | 17 (77.3) | 85 (68.0) |
| Q17_2_a | ...the impact on workforce needs | Disagree or strongly disagree | 31 (11.8) | 3 (5.5) | 4 (13.3) | 1 (3.3) | 3 (13.6) | 20 (16.0) |
|  |  | Neither agree nor disagree | 42 (16.0) | 10 (18.2) | 2 (6.7) | 9 (30.0) | 3 (13.6) | 18 (14.4) |
|  |  | Agree or strongly agree | 189 (72.1) | 42 (76.4) | 24 (80.0) | 20 (66.7) | 16 (72.7) | 87 (69.6) |
| Q17_3_a | ...the impact on job satisfaction | Disagree or strongly disagree | 51 (19.5) | 6 (10.9) | 6 (20.0) | 7 (23.3) | 5 (22.7) | 27 (21.6) |
|  |  | Neither agree nor disagree | 37 (14.1) | 10 (18.2) | 5 (16.7) | 7 (23.3) | 1 (4.5) | 14 (11.2) |
|  |  | Agree or strongly agree | 174 (66.4) | 39 (70.9) | 19 (63.3) | 16 (53.3) | 16 (72.7) | 84 (67.2) |
| Q17_4_a | ...training opportunities or career progression | Disagree or strongly disagree | 40 (15.3) | 7 (12.7) | 3 (10.0) | 4 (13.3) | 3 (13.6) | 23 (18.4) |
|  |  | Neither agree nor disagree | 23 (8.8) | 3 (5.5) | 4 (13.3) | 5 (16.7) | 2 (9.1) | 9 (7.2) |
|  |  | Agree or strongly agree | 199 (76.0) | 45 (81.8) | 23 (76.7) | 21 (70.0) | 17 (77.3) | 93 (74.4) |
| Q17_5_a | ...staff having a more relaxed/less rigorous approach to healthcare | Disagree or strongly disagree | 94 (35.9) | 20 (36.4) | 11 (36.7) | 9 (30.0) | 3 (13.6) | 51 (40.8) |
|  |  | Neither agree nor disagree | 62 (23.7) | 6 (10.9) | 10 (33.3) | 8 (26.7) | 5 (22.7) | 33 (26.4) |
|  |  | Agree or strongly agree | 106 (40.5) | 29 (52.7) | 9 (30.0) | 13 (43.3) | 14 (63.6) | 41 (32.8) |
| Q17_6_a | ...benchmarking performance of clinicians / graders against AI technology | Disagree or strongly disagree | 36 (13.7) | 4 (7.3) | 2 (6.7) | 5 (16.7) | 2 (9.1) | 23 (18.4) |
|  |  | Neither agree nor disagree | 57 (21.8) | 17 (30.9) | 7 (23.3) | 6 (20.0) | 6 (27.3) | 21 (16.8) |
|  |  | Agree or strongly agree | 169 (64.5) | 34 (61.8) | 21 (70.0) | 19 (63.3) | 14 (63.6) | 81 (64.8) |
| **Screening Experience** | | |  |  |  |  |  |  |
| Q19_1_a | ...adversely affect the relationship between patient and health care professionals within the DESP | Disagree or strongly disagree | 66 (25.2) | 9 (16.4) | 5 (16.7) | 12 (40.0) | 3 (13.6) | 37 (29.6) |
|  |  | Neither agree nor disagree | 58 (22.1) | 13 (23.6) | 9 (30.0) | 5 (16.7) | 6 (27.3) | 25 (20.0) |
|  |  | Agree or strongly agree | 138 (52.7) | 33 (60.0) | 16 (53.3) | 13 (43.3) | 13 (59.1) | 63 (50.4) |
| Q19_2_a | ...challenge the trust relationship between patients and health care professionals | Disagree or strongly disagree | 59 (22.5) | 10 (18.2) | 4 (13.3) | 9 (30.0) | 2 (9.1) | 34 (27.2) |
|  |  | Neither agree nor disagree | 45 (17.2) | 10 (18.2) | 4 (13.3) | 3 (10.0) | 4 (18.2) | 24 (19.2) |
|  |  | Agree or strongly agree | 158 (60.3) | 35 (63.6) | 22 (73.3) | 18 (60.0) | 16 (72.7) | 67 (53.6) |
| Q19_3_a | ...lead to a less personalised patient experience | Disagree or strongly disagree | 66 (25.2) | 8 (14.5) | 5 (16.7) | 13 (43.3) | 2 (9.1) | 38 (30.4) |
|  |  | Neither agree nor disagree | 25 (9.5) | 5 (9.1) | 1 (3.3) | 2 (6.7) | 4 (18.2) | 13 (10.4) |
|  |  | Agree or strongly agree | 171 (65.3) | 42 (76.4) | 24 (80.0) | 15 (50.0) | 16 (72.7) | 74 (59.2) |
| Q19_4_a | ...provide screening results at the time of image capture | Disagree or strongly disagree | 79 (30.2) | 17 (30.9) | 8 (26.7) | 8 (26.7) | 5 (22.7) | 41 (32.8) |
|  |  | Neither agree nor disagree | 74 (28.2) | 18 (32.7) | 5 (16.7) | 10 (33.3) | 7 (31.8) | 34 (27.2) |
|  |  | Agree or strongly agree | 109 (41.6) | 20 (36.4) | 17 (56.7) | 12 (40.0) | 10 (45.5) | 50 (40.0) |
| Q19_5_a | ...allow more time for communication between health care professionals and patients | Disagree or strongly disagree | 85 (32.4) | 21 (38.2) | 9 (30.0) | 4 (13.3) | 6 (27.3) | 45 (36.0) |
|  |  | Neither agree nor disagree | 76 (29.0) | 21 (38.2) | 7 (23.3) | 9 (30.0) | 6 (27.3) | 33 (26.4) |
|  |  | Agree or strongly agree | 101 (38.5) | 13 (23.6) | 14 (46.7) | 17 (56.7) | 10 (45.5) | 47 (37.6) |

## Table S3: Multivariable logistic regression results for people living with diabetes

| **Odds ratio for stand-alone questions (95% confidence interval)** | | | | | | | | | | | | |
| --- | --- | --- | --- | --- | --- | --- | --- | --- | --- | --- | --- | --- |
| **Characteristics** | **Q5** | **p-value** | **Q17** | **p-value** | **Q21** | **p-value** | **Q23** | **p-value** | **Q24_3_a** | **p-value** | **Q24_4_a** | **p-value** |
| **Site** *Base: South East London* |  |  |  |  |  |  |  |  |  |  |  |  |
| *North East London* | 1.08 (0.41,2.87) | 0.88 | 1.04 (0.82,1.32) | 0.76 | 1.01 (0.76,1.35) | 0.95 | 1.13 (0.89,1.44) | 0.31 | 0.97 (0.68,1.39) | 0.87 | 1.16 (0.89,1.51) | 0.26 |
| *Gloucester* | 1.00 (1.00,1.00) | - | 0.79 (0.39,1.61) | 0.52 | 1.24 (0.45,3.39) | 0.68 | 1.70 (0.80,3.60) | 0.17 | 0.53 (0.17,1.64) | 0.27 | 2.46 (0.91,6.67) | 0.08 |
| *Other* | 1.00 (1.00,1.00) | - | 1.27 (0.38,4.23) | 0.70 | 1.92 (0.38,9.69) | 0.43 | 1.08 (0.33,3.59) | 0.90 | 0.71 (0.17,3.02) | 0.64 | 1.00 (1.00,1.00) | - |
| **Age group**  *Base: ≥ 50 to < 60 years* |  |  |  |  |  |  |  |  |  |  |  |  |
| *< 40 years* | 0.35 (0.07,1.79) | 0.21 | 1.00 (0.56,1.76) | 0.99 | 0.70 (0.36,1.34) | 0.28 | 0.80 (0.46,1.39) | 0.42 | 0.57 (0.25,1.27) | 0.17 | 1.42 (0.72,2.79) | 0.31 |
| *≥ 40 to < 50 years* | 0.82 (0.17,3.94) | 0.81 | 0.81 (0.54,1.24) | 0.33 | 0.66 (0.41,1.06) | 0.08 | 1.02 (0.67,1.53) | 0.94 | 0.65 (0.36,1.17) | 0.15 | 1.16 (0.72,1.85) | 0.55 |
| *≥ 60 to < 70 years* | 1.06 (0.25,4.52) | 0.93 | 0.93 (0.68,1.29) | 0.68 | 0.98 (0.66,1.44) | 0.91 | 1.13 (0.82,1.56) | 0.45 | 0.44 (0.27,0.70) | 6.06E^-04^ | 0.97 (0.69,1.37) | 0.85 |
| *≥ 70 to ≤ 100 years* | 0.53 (0.10,2.82) | 0.46 | 0.78 (0.52,1.17) | 0.23 | 1.11 (0.68,1.82) | 0.67 | 1.21 (0.81,1.81) | 0.35 | 0.44 (0.24,0.80) | 7.17E^-03^ | 1.18 (0.77,1.83) | 0.44 |
| *missing* | 1.00 (1.00,1.00) | - | 1.10 (0.55,2.20) | 0.79 | 0.54 (0.27,1.10) | 0.09 | 1.01 (0.52,1.94) | 0.99 | 0.40 (0.12,1.41) | 0.15 | 1.10 (0.55,2.18) | 0.78 |
| **Sex**  *Base: Male* |  |  |  |  |  |  |  |  |  |  |  |  |
| *Female* | 0.74 (0.30,1.83) | 0.51 | 1.36 (1.09,1.70) | 0.01 | 0.52 (0.40,0.68) | 7.89E^-07^ | 0.65 (0.52,0.81) | 9.44E^-05^ | 1.11 (0.80,1.54) | 0.52 | 0.64 (0.51,0.81) | 1.93E^-04^ |
| *Prefer not to say* | 0.24 (0.02,3.12) | 0.28 | 0.27 (0.07,1.08) | 0.06 | 0.24 (0.06,0.94) | 0.04 | 0.56 (0.15,2.09) | 0.39 | 1.54 (0.27,8.75) | 0.62 | 0.71 (0.19,2.65) | 0.61 |
| **Ethnicity** *Base: White* |  |  |  |  |  |  |  |  |  |  |  |  |
| *Black* | 2.35 (0.47,11.70) | 0.30 | 1.77 (1.27,2.47) | 7.51E^-04^ | 0.71 (0.49,1.03) | 0.07 | 0.93 (0.67,1.28) | 0.65 | 0.22 (0.12,0.41) | 1.14E^-06^ | 0.67 (0.48,0.95) | 0.02 |
| *Asian* | 0.93 (0.29,3.02) | 0.90 | 2.32 (1.64,3.28) | 1.98E^-06^ | 1.19 (0.79,1.80) | 0.40 | 1.61 (1.14,2.25) | 6.06E^-03^ | 0.22 (0.11,0.44) | 1.06E^-05^ | 1.05 (0.73,1.51) | 0.80 |
| *Mixed/other/prefer not to say* | 1.44 (0.25,8.47) | 0.69 | 1.05 (0.66,1.65) | 0.85 | 0.44 (0.27,0.72) | 1.10E^-03^ | 0.65 (0.41,1.03) | 0.07 | 0.65 (0.33,1.28) | 0.21 | 0.56 (0.34,0.90) | 0.02 |
| **Highest Qualification** *Base: Degree or equivalent or above* |  |  |  |  |  |  |  |  |  |  |  |  |
| *No qualification or GCSE level qualification* | 1.15 (0.32,4.13) | 0.83 | 1.20 (0.92,1.57) | 0.19 | 1.00 (0.72,1.39) | 1.00 | 1.49 (1.14,1.96) | 3.99E^-03^ | 0.45 (0.30,0.69) | 1.99E^-04^ | 1.07 (0.80,1.43) | 0.65 |
| *A level or equivalent higher education qualification* | 0.59 (0.18,1.90) | 0.38 | 1.34 (1.01,1.77) | 0.04 | 0.91 (0.65,1.28) | 0.60 | 1.17 (0.89,1.54) | 0.26 | 0.63 (0.43,0.94) | 0.02 | 1.37 (1.00,1.86) | 0.05 |
| *Prefer not to say* | 0.57 (0.13,2.43) | 0.44 | 1.27 (0.80,2.02) | 0.30 | 0.62 (0.38,1.01) | 0.05 | 0.93 (0.59,1.45) | 0.74 | 0.39 (0.17,0.89) | 0.02 | 0.98 (0.61,1.58) | 0.95 |
| *other* | 1.00 (1.00,1.00) | - | 1.17 (0.39,3.53) | 0.77 | 0.92 (0.24,3.50) | 0.91 | 1.55 (0.50,4.81) | 0.45 | 0.86 (0.18,4.10) | 0.85 | 3.29 (0.71,15.33) | 0.13 |
| **Employment status**  *Base: Retired* |  |  |  |  |  |  |  |  |  |  |  |  |
| *In full-time employment* | 1.67 (0.33,8.48) | 0.53 | 1.02 (0.73,1.43) | 0.91 | 1.02 (0.68,1.53) | 0.94 | 1.24 (0.88,1.73) | 0.21 | 0.99 (0.59,1.65) | 0.96 | 1.31 (0.91,1.89) | 0.15 |
| *In part-time employment* | 2.08 (0.21,20.39) | 0.53 | 1.19 (0.77,1.83) | 0.44 | 1.12 (0.67,1.87) | 0.67 | 1.37 (0.89,2.10) | 0.15 | 0.66 (0.33,1.33) | 0.25 | 0.95 (0.61,1.49) | 0.83 |
| *Not working (seeking work, looking after home/family, student, sickness/disability)* | 0.74 (0.14,3.76) | 0.71 | 0.68 (0.46,1.01) | 0.06 | 1.14 (0.70,1.84) | 0.61 | 1.27 (0.85,1.90) | 0.24 | 0.54 (0.27,1.07) | 0.08 | 0.93 (0.61,1.43) | 0.75 |
| *Prefer not to say/other* | 0.31 (0.06,1.65) | 0.17 | 0.78 (0.44,1.36) | 0.38 | 0.59 (0.32,1.08) | 0.08 | 0.87 (0.50,1.51) | 0.61 | 1.05 (0.45,2.44) | 0.91 | 0.55 (0.31,0.98) | 0.04 |
| **Townsend score**  *Base: >6* |  |  |  |  |  |  |  |  |  |  |  |  |
| *≤ -0.5 (least deprived)* | 6.04 (0.67,54.79) | 0.11 | 1.13 (0.79,1.62) | 0.51 | 1.02 (0.66,1.57) | 0.94 | 0.73 (0.51,1.04) | 0.08 | 1.03 (0.60,1.77) | 0.91 | 0.66 (0.45,0.97) | 0.03 |
| *>-0.5 to ≤ 2* | 2.52 (0.57,11.14) | 0.22 | 0.87 (0.62,1.22) | 0.41 | 0.88 (0.59,1.31) | 0.53 | 0.79 (0.56,1.10) | 0.16 | 0.98 (0.57,1.66) | 0.93 | 0.85 (0.59,1.23) | 0.40 |
| *>2 to ≤ 4* | 1.37 (0.39,4.84) | 0.62 | 0.95 (0.69,1.32) | 0.78 | 1.31 (0.88,1.95) | 0.18 | 0.79 (0.57,1.10) | 0.16 | 1.25 (0.76,2.07) | 0.38 | 0.94 (0.66,1.34) | 0.74 |
| *>4 to ≤ 6 (most deprived)* | 1.01 (0.30,3.37) | 0.99 | 1.05 (0.75,1.46) | 0.80 | 1.03 (0.70,1.52) | 0.89 | 0.90 (0.64,1.25) | 0.53 | 1.50 (0.91,2.49) | 0.11 | 0.90 (0.62,1.29) | 0.56 |
| **About you** |  |  |  |  |  |  |  |  |  |  |  |  |
| **Diabetes Type** *Base: Type 2* |  |  |  |  |  |  |  |  |  |  |  |  |
| *Type 1* | 0.48 (0.11,2.08) | 0.33 | 0.82 (0.56,1.20) | 0.31 | 0.98 (0.62,1.56) | 0.93 | 0.94 (0.64,1.38) | 0.74 | 1.56 (0.93,2.60) | 0.09 | 1.13 (0.73,1.74) | 0.58 |
| *Other/don't know* | 0.31 (0.10,0.96) | 0.04 | 0.90 (0.59,1.35) | 0.60 | 0.90 (0.56,1.46) | 0.68 | 0.90 (0.60,1.36) | 0.62 | 1.59 (0.88,2.86) | 0.12 | 1.09 (0.70,1.69) | 0.71 |
| **Duration of diabetes**  *Base: 0-5 years* |  |  |  |  |  |  |  |  |  |  |  |  |
| *6 - 10 years* | 1.04 (0.31,3.48) | 0.95 | 0.93 (0.70,1.23) | 0.60 | 1.23 (0.88,1.74) | 0.23 | 1.18 (0.88,1.56) | 0.26 | 0.88 (0.57,1.37) | 0.57 | 0.94 (0.69,1.27) | 0.68 |
| *11 - 15 years* | 1.67 (0.32,8.75) | 0.55 | 1.18 (0.85,1.64) | 0.32 | 1.30 (0.88,1.92) | 0.19 | 1.39 (1.01,1.92) | 0.05 | 1.14 (0.71,1.85) | 0.59 | 1.02 (0.72,1.45) | 0.91 |
| *16+ years* | 1.32 (0.34,5.16) | 0.68 | 1.02 (0.76,1.39) | 0.87 | 1.16 (0.81,1.67) | 0.42 | 1.23 (0.91,1.67) | 0.17 | 1.00 (0.63,1.57) | 0.99 | 1.00 (0.72,1.39) | 0.99 |
| *missing* | 1.00 (1.00,1.00) | - | 0.78 (0.25,2.43) | 0.67 | 1.86 (0.38,9.10) | 0.44 | 2.23 (0.64,7.76) | 0.21 | 0.57 (0.07,4.99) | 0.61 | 1.18 (0.34,4.13) | 0.79 |
| **Last attended DESP**  *Base: Within last two years* |  |  |  |  |  |  |  |  |  |  |  |  |
| *NOT within last two years (including first time attender/do not remember/missing)* | 0.30 (0.10,0.85) | 0.02 | 0.89 (0.63,1.26) | 0.53 | 1.25 (0.83,1.89) | 0.29 | 1.34 (0.94,1.89) | 0.10 | 0.70 (0.40,1.22) | 0.20 | 1.14 (0.78,1.66) | 0.50 |
| **Technology in daily life** *Base: 5 or more applications* |  |  |  |  |  |  |  |  |  |  |  |  |
| *less than 5 applications* | 0.74 (0.29,1.87) | 0.52 | 1.26 (1.01,1.58) | 0.04 | 0.69 (0.53,0.90) | 7.14E^-03^ | 1.16 (0.92,1.45) | 0.20 | 0.71 (0.49,1.01) | 0.06 | 0.53 (0.42,0.67) | 2.00E^-07^ |
| **Use of health-based apps**  *Base: yes* |  |  |  |  |  |  |  |  |  |  |  |  |
| *no* | 1.27 (0.52,3.15) | 0.60 | 0.94 (0.76,1.17) | 0.60 | 0.65 (0.50,0.84) | 1.15E^-03^ | 0.70 (0.56,0.87) | 1.28E^-03^ | 0.92 (0.66,1.28) | 0.61 | 0.77 (0.61,0.97) | 0.03 |

Q5: ''The Diabetic Eye Screening Programme is important in monitoring my condition.'

Q17: “'I am concerned that not knowing how AI works is a barrier to people living with diabetes accepting this technology for diabetic eye screening”

Q21: “If my eye images were processed by a computer or AI technology, I would be happy for these data to be securely stored under the control of the NHS and used for research to do with healthcare evaluation or improvement”

Q23: “I believe AI could detect diabetic eye disease equally well in people of different ethnic groups and different ages”

Q24_3_a: “It would be important for me to receive the results of my eye assessment on the day”

Q24_4_a: “I would be happy to receive results from my eye assessment via a text message or email rather than in a letter”

## Table S4: Multivariable logistic regression results for health care practitioners

| **Odds ratio for stand-alone questions (95% confidence interval) [HCP]** | | | | | | | | |
| --- | --- | --- | --- | --- | --- | --- | --- | --- |
| **Characteristic** | **Q11** | **p-value** | **Q15** | **p-value** | **Q16_5_a** | **p-value** | **Q18** | **p-value** |
| **Site** *Base: BARS* |  |  |  |  |  |  |  |  |
| *North East London* | 0.32 (0.13,0.77) | 0.01 | 0.42 (0.15,1.19) | 0.10 | 1.73 (0.49,6.07) | 0.39 | 0.66 (0.26,1.71) | 0.39 |
| *South East London* | 0.75 (0.28,1.98) | 0.56 | 1.10 (0.39,3.11) | 0.86 | 3.27 (0.90,11.85) | 0.07 | 0.59 (0.21,1.63) | 0.31 |
| *Gloucester* | 0.99 (0.41,2.40) | 0.98 | 0.30 (0.09,0.97) | 0.04 | 0.77 (0.22,2.72) | 0.68 | 1.53 (0.52,4.46) | 0.44 |
| *Tyneside* | 0.92 (0.31,2.69) | 0.88 | 1.77 (0.54,5.81) | 0.35 | 6.81 (1.67,27.72) | 0.01 | 3.23 (0.59,17.58) | 0.18 |
| **Age group** *Base: ≥ 50 to < 60 years* |  |  |  |  |  |  |  |  |
| *< 30 years* | 2.67 (0.91,7.89) | 0.07 | 4.46 (1.35,14.72) | 0.01 | 0.92 (0.23,3.62) | 0.90 | 0.84 (0.25,2.76) | 0.77 |
| *≥ 30 to < 40 years* | 1.48 (0.68,3.20) | 0.32 | 1.75 (0.69,4.46) | 0.24 | 0.98 (0.34,2.84) | 0.96 | 1.12 (0.45,2.80) | 0.80 |
| *≥ 40 to < 50 years* | 1.26 (0.59,2.70) | 0.55 | 2.12 (0.82,5.50) | 0.12 | 1.19 (0.40,3.53) | 0.75 | 0.46 (0.20,1.08) | 0.07 |
| *60+ years* | 1.27 (0.50,3.27) | 0.61 | 7.28 (2.39,22.22) | 4.87E^-04^ | 1.68 (0.43,6.47) | 0.45 | 1.21 (0.37,3.92) | 0.75 |
| **Sex** *Base: Male* |  |  |  |  |  |  |  |  |
| *Female* | 1.17 (0.64,2.12) | 0.61 | 0.86 (0.43,1.73) | 0.68 | 1.66 (0.69,3.99) | 0.26 | 0.80 (0.40,1.62) | 0.54 |
| *Prefer not to say* | 0.41 (0.02,10.58) | 0.59 | 1.00 (1.00,1.00) | - | 1.00 (1.00,1.00) | - | 0.16 (0.01,3.73) | 0.25 |
| **Ethnicity** *Base: White* |  |  |  |  |  |  |  |  |
| *Black* | 1.75 (0.26,11.92) | 0.57 | 0.09 (0.01,1.15) | 0.06 | 6.43 (0.82,50.58) | 0.08 | 0.62 (0.10,3.71) | 0.60 |
| *Asian* | 2.08 (0.83,5.22) | 0.12 | 0.21 (0.07,0.65) | 0.01 | 2.20 (0.69,7.07) | 0.18 | 1.36 (0.54,3.42) | 0.52 |
| *Mixed/other/prefer not to say* | 1.60 (0.50,5.06) | 0.43 | 0.98 (0.26,3.60) | 0.97 | 3.34 (0.79,14.06) | 0.10 | 5.78 (1.02,32.80) | 0.05 |
| **Townsend score** *Base: ≥ -0.5 to <2* |  |  |  |  |  |  |  |  |
| *<-2 (least deprived)* | 0.78 (0.34,1.80) | 0.57 | 0.89 (0.34,2.32) | 0.81 | 5.75 (1.68,19.66) | 0.01 | 0.70 (0.26,1.84) | 0.46 |
| *≥ -2 to <-0.5* | 1.18 (0.50,2.79) | 0.70 | 0.35 (0.12,0.97) | 0.04 | 2.79 (0.83,9.41) | 0.10 | 0.87 (0.30,2.51) | 0.80 |
| *≥ 2 to <4* | 0.79 (0.32,1.95) | 0.61 | 1.84 (0.69,4.87) | 0.22 | 0.68 (0.14,3.20) | 0.63 | 0.34 (0.12,0.93) | 0.03 |
| *≥ 4.0 (most deprived)* | 1.42 (0.55,3.64) | 0.47 | 3.98 (1.33,11.86) | 0.01 | 0.98 (0.26,3.72) | 0.97 | 0.57 (0.20,1.63) | 0.30 |
| *missing* | 0.99 (0.08,12.75) | 0.99 | 0.30 (0.02,5.37) | 0.41 | 15.13 (0.92,250.25) | 0.06 | 1.00 (1.00,1.00) | - |
| **Role** *Base: Screener, grader, photographer or optometrist* |  |  |  |  |  |  |  |  |
| *Clinical Lead or management position* | 1.30 (0.65,2.62) | 0.46 | 1.07 (0.49,2.34) | 0.87 | 0.29 (0.10,0.82) | 0.02 | 0.49 (0.22,1.06) | 0.07 |
| *Senior screener /grader* | 1.09 (0.53,2.27) | 0.81 | 0.27 (0.10,0.69) | 0.01 | 0.28 (0.10,0.83) | 0.02 | 0.86 (0.37,1.96) | 0.71 |
| *Administrator, fail safe officer, IT officer or other* | 1.08 (0.43,2.75) | 0.87 | 1.82 (0.67,4.96) | 0.24 | 0.16 (0.04,0.64) | 0.01 | 0.59 (0.20,1.77) | 0.35 |
| **Length of role**  *Base: Less than 5 years* |  |  |  |  |  |  |  |  |
| *5 to less than 10 years* | 1.77 (0.86,3.62) | 0.12 | 1.28 (0.59,2.78) | 0.53 | 1.60 (0.63,4.04) | 0.32 | 0.70 (0.33,1.52) | 0.37 |
| *10 years or more* | 1.04 (0.52,2.06) | 0.92 | 0.44 (0.19,1.02) | 0.06 | 0.43 (0.15,1.24) | 0.12 | 0.99 (0.44,2.21) | 0.98 |
| **Technology in daily life** *Base: 5 or more applications* |  |  |  |  |  |  |  |  |
| *less than 5 applications* | 0.88 (0.36,2.14) | 0.78 | 0.73 (0.25,2.08) | 0.55 | 1.22 (0.40,3.78) | 0.73 | 1.07 (0.36,3.16) | 0.91 |
| **Use of health-based apps** *Base: yes* |  |  |  |  |  |  |  |  |
| *no* | 0.76 (0.44,1.32) | 0.33 | 0.80 (0.42,1.53) | 0.50 | 1.13 (0.53,2.44) | 0.75 | 0.78 (0.42,1.47) | 0.44 |

Q11: “A lack of transparency in how AI works is a barrier to health care professionals accepting this technology for use within the DESP”

Q15: “I believe AI could detect diabetic eye disease equally well in people of different ethnic groups and different ages”

Q16_5_a: “...wholly replace human grading of retinal images for detection of diabetic eye disease within the DESP”

Q18: “Further training would be required for staff working in the DESP if AI systems for analysing retinal images were to be implemented”

## Table S5: Multivariable Linear regression results among health app users (people living with diabetes)

| **Differences in scores compared to base group (95% confidence interval)** | | | | | | | | | | |
| --- | --- | --- | --- | --- | --- | --- | --- | --- | --- | --- |
| **Characteristic** | **General score** | **p-value** | **Efficiency score** | **p-value** | **Data Security score** | **p-value** | **Trust score** | **p-value** | **Screening experience** | **p-value** |
| **Site** *Base: South East London* |  |  |  |  |  |  |  |  |  |  |
| *North East London* | 0.07  (-0.04,0.19) | 0.19 | -0.04  (-0.15,0.08) | 0.54 | -0.05  (-0.18,0.08) | 0.48 | -0.03  (-0.13,0.07) | 0.54 | -0.04  (-0.13,0.05) | 0.33 |
| *Gloucester* | 0.25  (-0.07,0.56) | 0.12 | 0.14  (-0.17,0.46) | 0.37 | 0.44  (0.07,0.81) | 0.02 | 0.19  (-0.08,0.46) | 0.17 | 0.16  (-0.10,0.41) | 0.23 |
| *Other* | 0.08  (-0.35,0.52) | 0.71 | 0.07  (-0.37,0.51) | 0.75 | 0.04  (-0.48,0.56) | 0.88 | 0.18  (-0.20,0.56) | 0.35 | -0.05  (-0.41,0.31) | 0.79 |
| **Age group** *Base: ≥ 50 to < 60 year*s |  |  |  |  |  |  |  |  |  |  |
| *< 40 years* | -0.01  (-0.25,0.23) | 0.92 | 0.03  (-0.21,0.27) | 0.79 | 0.13  (-0.15,0.42) | 0.35 | 0.12  (-0.09,0.32) | 0.27 | -0.05  (-0.24,0.14) | 0.60 |
| *≥ 40 to < 50 years* | 0.07  (-0.10,0.25) | 0.40 | 0.00  (-0.18,0.17) | 0.98 | 0.23  (0.03,0.44)* | 0.03 | 0.01  (-0.14,0.16) | 0.87 | 0.03  (-0.11,0.17) | 0.68 |
| *≥ 60 to < 70 years* | 0.07  (-0.07,0.21) | 0.35 | 0.05  (-0.09,0.20) | 0.46 | 0.18  (0.01,0.35)* | 0.04 | 0.00  (0.00,0.00) | 0.50 | 0.05  (-0.06,0.17) | 0.39 |
| *≥ 70 to ≤ 100 years* | 0.02  (-0.16,0.21) | 0.80 | 0.03  (-0.15,0.22) | 0.72 | -0.01  (-0.22,0.21) | 0.95 | 0.09  (-0.07,0.25) | 0.26 | 0.03  (-0.12,0.18) | 0.67 |
| *missing* | 0.06  (-0.23,0.35) | 0.68 | -0.27  (-0.56,0.02) | 0.07 | -0.05  (-0.40,0.29) | 0.76 | 0.17  (-0.09,0.42) | 0.20 | -0.13  (-0.37,0.11) | 0.28 |
| **Sex** *Base: Male* |  |  |  |  |  |  |  |  |  |  |
| *Female* | -0.16  (-0.26,-0.07) | 1.16E-03 | -0.21  (-0.31,-0.11) | 5.00E-05 | -0.19  (-0.31,-0.07) | 1.65E-03 | -0.17  (-0.26,-0.09) | 7.45E-05 | -0.16  (-0.24,-0.08) | 1.23E-04 |
| *Prefer not to say* | 0.17  (-0.36,0.71) | 0.52 | -0.30  (-0.84,0.23) | 0.27 | 0.23  (-0.41,0.86) | 0.48 | 0.00  (-0.46,0.46) | 1.00 | 0.01  (-0.43,0.44) | 0.98 |
| **Ethnicity**  *Base: White* |  |  |  |  |  |  |  |  |  |  |
| *Black* | -0.07  (-0.22,0.07) | 0.34 | -0.17  (-0.31,-0.02) | 0.03 | -0.16  (-0.33,0.02) | 0.08 | -0.10  (-0.22,0.03) | 0.13 | -0.46  (-0.58,-0.34) | 3.24E-14 |
| *Asian* | -0.06  (-0.22,0.09) | 0.42 | -0.13  (-0.29,0.02) | 0.09 | -0.30  (-0.48,-0.11) | 1.47E-03 | 0.03  (-0.10,0.17) | 0.63 | -0.31  (-0.44,-0.19) | 1.04E-06 |
| *Mixed/other/prefer not to say* | -0.11  (-0.31,0.08) | 0.26 | -0.12  (-0.32,0.08) | 0.25 | -0.06  (-0.30,0.17) | 0.59 | -0.15  (-0.32,0.02) | 0.08 | -0.15  (-0.31,0.01) | 0.06 |
| **Highest Qualification**  *Base: Degree or equivalent or above* |  |  |  |  |  |  |  |  |  |  |
| *No qualification or GCSE level qualification* | -0.08  (-0.20,0.05) | 0.23 | -0.12  (-0.25,0.00) | 0.06 | -0.08  (-0.24,0.07) | 0.28 | 0.02  (-0.09,0.13) | 0.76 | -0.33  (-0.43,-0.23)ⱡ | 3.72E-10 |
| *A level or equivalent higher education qualification* | -0.08  (-0.20,0.05) | 0.23 | -0.14  (-0.26,-0.02) | 0.03 | -0.11  (-0.26,0.03) | 0.13 | 0.05  (-0.05,0.16) | 0.33 | -0.22  (-0.32,-0.12)ⱡ | 1.83E-05 |
| *Prefer not to say* | -0.33  (-0.56,-0.11) | 3.49E-03 | -0.33  (-0.55,-0.10) | 4.01E-03 | -0.20  (-0.47,0.06) | 0.13 | -0.18  (-0.37,0.01) | 0.07 | -0.39  (-0.57,-0.21)ⱡ | 2.64E-05 |
| *other* | -0.16  (-0.86,0.55) | 0.66 | 0.18  (-0.53,0.89) | 0.62 | 0.47  (-0.37,1.31) | 0.27 | -0.20  (-0.81,0.41) | 0.51 | -0.18  (-0.75,0.40) | 0.55 |
| **Employment status** *Base: Retired* |  |  |  |  |  |  |  |  |  |  |
| *In full-time employment* | 0.07  (-0.09,0.22) | 0.41 | 0.08  (-0.07,0.24) | 0.31 | -0.09  (-0.27,0.09) | 0.34 | 0.01  (-0.12,0.15) | 0.85 | 0.04  (-0.09,0.17) | 0.54 |
| *In part-time employment* | 0.14  (-0.05,0.33) | 0.14 | 0.10  (-0.09,0.29) | 0.29 | -0.07  (-0.29,0.15) | 0.54 | 0.04  (-0.13,0.20) | 0.65 | 0.02  (-0.13,0.17) | 0.80 |
| *Not working (seeking work, looking after home/family, student, sickness/disability)* | 0.06  (-0.12,0.25) | 0.50 | -0.01  (-0.19,0.17) | 0.92 | -0.08  (-0.29,0.14) | 0.50 | 0.03  (-0.13,0.19) | 0.68 | -0.01  (-0.16,0.14) | 0.91 |
| *Prefer not to say/other* | -0.09  (-0.34,0.16) | 0.47 | -0.04  (-0.29,0.21) | 0.76 | -0.21  (-0.51,0.08) | 0.16 | 0.04  (-0.18,0.26) | 0.71 | -0.11  (-0.32,0.09) | 0.28 |
| **Townsend score** *Base: >6* |  |  |  |  |  |  |  |  |  |  |
| *≤ -0.5* | 0.05  (-0.11,0.22) | 0.51 | 0.05  (-0.12,0.21) | 0.58 | 0.01  (-0.18,0.20) | 0.92 | -0.09  (-0.23,0.05) | 0.21 | 0.00  (-0.13,0.13) | 0.96 |
| *> -0.5 to ≤ 2* | 0.06  (-0.10,0.22) | 0.47 | 0.17  (0.01,0.33) | 0.04 | -0.05  (-0.24,0.14) | 0.58 | -0.04  (-0.18,0.09) | 0.54 | 0.05  (-0.08,0.18) | 0.47 |
| *>2 to ≤ 4* | 0.03  (-0.12,0.18) | 0.71 | 0.03  (-0.12,0.18) | 0.68 | -0.01  (-0.18,0.17) | 0.94 | -0.01  (-0.14,0.12) | 0.88 | 0.07  (-0.05,0.19) | 0.25 |
| *> 4 to ≤ 6* | 0.13  (-0.02,0.28) | 0.08 | 0.15  (0.00,0.30) | 0.05 | 0.02  (-0.16,0.20) | 0.82 | 0.07  (-0.06,0.20) | 0.26 | 0.11  (-0.02,0.23) | 0.09 |
| **About you** |  |  |  |  |  |  |  |  |  |  |
| **Diabetes Type** *Base: Type 2* |  |  |  |  |  |  |  |  |  |  |
| *Type 1* | 0.12  (-0.05,0.30) | 0.16 | 0.11  (-0.07,0.28) | 0.23 | 0.07  (-0.14,0.27) | 0.52 | 0.06  (-0.09,0.21) | 0.44 | 0.16  (0.02,0.30) | 0.03 |
| *Other/don't know* | -0.05  (-0.25,0.15) | 0.61 | -0.12  (-0.31,0.08) | 0.25 | 0.12  (-0.12,0.35) | 0.33 | 0.02  (-0.15,0.19) | 0.85 | 0.14  (-0.02,0.30) | 0.09 |
| **Duration of diabetes** *Base: 0-5 years* |  |  |  |  |  |  |  |  |  |  |
| *6 - 10 years* | 0.17  (0.04,0.29) | 0.01 | 0.13  (0.01,0.26) | 0.04 | -0.04  (-0.19,0.12) | 0.63 | 0.05  (-0.06,0.16) | 0.39 | 0.09  (-0.01,0.20) | 0.08 |
| *11 - 15 years* | 0.28  (0.13,0.42)ⱡ | 2.12E-04 | 0.21  (0.07,0.36) | 4.43E-03 | 0.01  (-0.16,0.18) | 0.90 | 0.09  (-0.04,0.22) | 0.16 | 0.10  (-0.02,0.22) | 0.09 |
| *16+ years* | 0.11  (-0.03,0.26) | 0.11 | 0.17  (0.03,0.31) | 0.02 | 0.02  (-0.15,0.19) | 0.78 | 0.04  (-0.08,0.16) | 0.52 | 0.11  (-0.01,0.22) | 0.07 |
| *missing* | 0.45  (-0.09,1.00) | 0.10 | 0.66  (0.11,1.20) | 0.02 | 0.07  (-0.58,0.71) | 0.84 | 0.35  (-0.12,0.82) | 0.14 | 0.01  (-0.44,0.45) | 0.97 |
| **Last attended DESP**  *Base: Within last two years* |  |  |  |  |  |  |  |  |  |  |
| *NOT within last two years (including first time attender/do not remember/missing)* | 0.06  (-0.09,0.21) | 0.45 | 0.08  (-0.07,0.24) | 0.29 | -0.05  (-0.23,0.13) | 0.59 | 0.02  (-0.12,0.15) | 0.80 | 0.08  (-0.05,0.21) | 0.21 |
| **Technology in daily life:** *Base: 5 or more applications* |  |  |  |  |  |  |  |  |  |  |
| *less than 5 applications* | 0.01  (-0.10,0.12) | 0.87 | -0.10  (-0.20,0.01) | 0.07 | -0.14  (-0.27,-0.02) | 0.03 | 0.03  (-0.07,0.12) | 0.59 | -0.18  (-0.27,-0.09) | 4.82E-05 |
| **Trust in health-based results from used applications** *Base: Sometimes* |  |  |  |  |  |  |  |  |  |  |
| *Unsure, never or rarely* | -0.19  (-0.39,0.02) | 0.08 | -0.09  (-0.30,0.12) | 0.40 | -0.06  (-0.31,0.19) | 0.63 | -0.10  (-0.28,0.08) | 0.26 | 0.01  (-0.15,0.18) | 0.86 |
| *Often* | 0.22  (0.11,0.33) | 1.38E-04 | 0.18  (0.07,0.30) | 1.60E-03 | 0.04  (-0.09,0.18) | 0.52 | 0.17  (0.08,0.27) | 4.71E-04 | 0.05  (-0.04,0.15) | 0.25 |
| *Very often or always* | 0.45  (0.30,0.59) | 2.30E-09 | 0.33  (0.18,0.47) | 1.45E-05 | 0.08  (-0.10,0.25) | 0.39 | 0.26  (0.14,0.39) | 5.33E-05 | 0.03  (-0.09,0.15) | 0.58 |

## Table S6: Multivariable logistic regression results among health app users (people living with diabetes)

| **Odds ratio for stand-alone questions (95% confidence interval)** | | | | | | | | | | | | |
| --- | --- | --- | --- | --- | --- | --- | --- | --- | --- | --- | --- | --- |
| **Characteristics** | **Q5** | **p-value** | **Q17** | **p-value** | **Q21** | **p-value** | **Q23** | **p-value** | **Q24_3_a** | **p-value** | **Q24_4_a** | **p-value** |
| **Site** *Base: South East London* |  |  |  |  |  |  |  |  |  |  |  |  |
| *North East London* | 0.64 (0.16,2.64) | 0.54 | 0.96 (0.68,1.35) | 0.82 | 1.12 (0.72,1.73) | 0.62 | 1.11 (0.79,1.57) | 0.54 | 0.93 (0.57,1.52) | 0.78 | 1.25 (0.85,1.82) | 0.25 |
| *Gloucester* | 1.00 (1.00,1.00) | - | 0.90 (0.35,2.29) | 0.83 | 1.44 (0.37,5.54) | 0.60 | 2.14 (0.77,6.00) | 0.15 | 0.62 (0.16,2.42) | 0.49 | 3.05 (0.82,11.40) | 0.10 |
| *Other* | 1.00 (1.00,1.00) | - | 1.82 (0.47,7.04) | 0.39 | 2.30 (0.27,19.87) | 0.45 | 1.10 (0.30,4.01) | 0.89 | 0.63 (0.13,2.96) | 0.56 | 1.00 (1.00,1.00) | - |
| **Age group**  *Base: ≥ 50 to < 60 years* |  |  |  |  |  |  |  |  |  |  |  |  |
| *< 40 years* | 0.08 (0.01,0.92) | 0.04 | 1.55 (0.74,3.24) | 0.25 | 0.72 (0.29,1.75) | 0.47 | 0.72 (0.35,1.47) | 0.36 | 0.55 (0.20,1.50) | 0.25 | 1.06 (0.44,2.55) | 0.90 |
| *≥ 40 to < 50 years* | 0.60 (0.04,9.43) | 0.71 | 0.80 (0.47,1.37) | 0.42 | 0.53 (0.28,0.99) | 0.05 | 1.01 (0.59,1.72) | 0.98 | 0.92 (0.45,1.89) | 0.82 | 0.86 (0.47,1.59) | 0.63 |
| *≥ 60 to < 70 years* | 0.80 (0.07,9.03) | 0.86 | 1.17 (0.75,1.81) | 0.50 | 0.90 (0.51,1.59) | 0.72 | 1.06 (0.68,1.65) | 0.79 | 0.65 (0.35,1.22) | 0.18 | 0.72 (0.45,1.17) | 0.18 |
| *≥ 70 to ≤ 100 years* | 0.22 (0.02,2.92) | 0.25 | 0.77 (0.44,1.36) | 0.37 | 0.81 (0.39,1.70) | 0.58 | 0.94 (0.54,1.66) | 0.84 | 0.76 (0.34,1.68) | 0.49 | 1.37 (0.74,2.54) | 0.31 |
| *missing* | 1.00 (1.00,1.00) | - | 0.98 (0.38,2.52) | 0.97 | 0.35 (0.13,0.93) | 0.04 | 1.22 (0.49,3.03) | 0.67 | 0.66 (0.14,3.20) | 0.60 | 0.74 (0.30,1.85) | 0.52 |
| **Sex**  *Base: Male* |  |  |  |  |  |  |  |  |  |  |  |  |
| *Female* | 1.21 (0.29,5.11) | 0.79 | 1.71 (1.25,2.32) | 6.69E^-04^ | 0.54 (0.37,0.80) | 2.12E^-03^ | 0.61 (0.45,0.82) | 1.26E^-03^ | 1.27 (0.82,1.96) | 0.28 | 0.69 (0.50,0.96) | 0.03 |
| *Prefer not to say* | 0.13 (0.00,3.25) | 0.21 | 0.08 (0.01,0.81) | 0.03 | 0.32 (0.06,1.77) | 0.19 | 0.96 (0.18,5.04) | 0.96 | 0.80 (0.07,8.51) | 0.85 | 0.44 (0.08,2.40) | 0.34 |
| **Ethnicity** *Base: White* |  |  |  |  |  |  |  |  |  |  |  |  |
| *Black* | 1.00 (1.00,1.00) | - | 1.94 (1.23,3.08) | 4.61E^-03^ | 0.61 (0.35,1.07) | 0.08 | 0.73 (0.46,1.15) | 0.17 | 0.19 (0.08,0.42) | 5.67E^-05^ | 0.56 (0.34,0.90) | 0.02 |
| *Asian* | 0.78 (0.13,4.78) | 0.79 | 2.73 (1.65,4.53) | 9.22E^-05^ | 0.78 (0.42,1.46) | 0.44 | 1.48 (0.90,2.44) | 0.13 | 0.29 (0.13,0.65) | 2.91E^-03^ | 0.87 (0.51,1.47) | 0.60 |
| *Mixed/other/prefer not to say* | 1.63 (0.08,31.89) | 0.75 | 1.50 (0.82,2.75) | 0.19 | 0.47 (0.24,0.94) | 0.03 | 0.75 (0.41,1.37) | 0.35 | 0.55 (0.22,1.34) | 0.19 | 0.52 (0.27,0.97) | 0.04 |
| **Highest Qualification** *Base: Degree or equivalent or above* |  |  |  |  |  |  |  |  |  |  |  |  |
| *No qualification or GCSE level qualification* | 1.23 (0.13,11.89) | 0.86 | 1.54 (1.04,2.27) | 0.03 | 0.65 (0.39,1.08) | 0.10 | 1.08 (0.73,1.61) | 0.70 | 0.38 (0.21,0.67) | 9.63E^-04^ | 0.83 (0.55,1.26) | 0.39 |
| *A level or equivalent higher education qualification* | 0.22 (0.03,1.40) | 0.11 | 1.58 (1.08,2.31) | 0.02 | 0.76 (0.47,1.24) | 0.28 | 1.06 (0.73,1.54) | 0.77 | 0.56 (0.33,0.95) | 0.03 | 1.38 (0.90,2.11) | 0.14 |
| *Prefer not to say* | 0.12 (0.01,1.17) | 0.07 | 2.41 (1.11,5.22) | 0.03 | 0.36 (0.17,0.78) | 9.40E^-03^ | 0.41 (0.21,0.82) | 0.01 | 0.18 (0.04,0.86) | 0.03 | 1.27 (0.60,2.68) | 0.53 |
| *other* | 1.00 (1.00,1.00) | - | 0.97 (0.11,8.14) | 0.97 | 0.34 (0.03,3.62) | 0.37 | 1.33 (0.13,13.73) | 0.81 | 3.98 (0.45,35.66) | 0.22 | 1.20 (0.11,12.79) | 0.88 |
| **Employment status**  *Base: Retired* |  |  |  |  |  |  |  |  |  |  |  |  |
| *In full-time employment* | 1.46 (0.13,16.16) | 0.76 | 1.27 (0.78,2.05) | 0.33 | 0.73 (0.39,1.36) | 0.32 | 1.13 (0.70,1.82) | 0.62 | 1.08 (0.54,2.13) | 0.83 | 1.73 (1.03,2.90) | 0.04 |
| *In part-time employment* | 0.85 (0.05,13.74) | 0.91 | 1.21 (0.67,2.18) | 0.53 | 1.14 (0.52,2.47) | 0.75 | 1.54 (0.85,2.79) | 0.16 | 0.73 (0.30,1.78) | 0.48 | 1.29 (0.70,2.38) | 0.42 |
| *Not working (seeking work, looking after home/family, student, sickness/disability)* | 1.00 (1.00,1.00) | - | 0.59 (0.33,1.03) | 0.06 | 0.96 (0.45,2.02) | 0.91 | 1.10 (0.62,1.96) | 0.73 | 0.55 (0.22,1.39) | 0.21 | 1.05 (0.58,1.91) | 0.87 |
| *Prefer not to say/other* | 0.52 (0.04,6.51) | 0.61 | 0.86 (0.40,1.87) | 0.71 | 0.39 (0.16,0.94) | 0.04 | 0.82 (0.38,1.77) | 0.61 | 1.97 (0.73,5.32) | 0.18 | 0.73 (0.33,1.60) | 0.43 |
| **Townsend score**  *Base: >6* |  |  |  |  |  |  |  |  |  |  |  |  |
| *≤ -0.5 (least deprived)* | 4.31 (0.29,63.11) | 0.29 | 1.27 (0.77,2.09) | 0.36 | 1.02 (0.54,1.92) | 0.94 | 0.87 (0.53,1.44) | 0.60 | 0.65 (0.32,1.34) | 0.24 | 0.60 (0.35,1.03) | 0.06 |
| *>-0.5 to ≤ 2* | 1.16 (0.16,8.61) | 0.88 | 0.72 (0.44,1.18) | 0.19 | 1.32 (0.71,2.47) | 0.38 | 1.30 (0.79,2.16) | 0.30 | 0.75 (0.36,1.55) | 0.44 | 0.89 (0.52,1.54) | 0.68 |
| *>2 to ≤ 4* | 1.08 (0.14,8.56) | 0.94 | 0.78 (0.49,1.23) | 0.29 | 1.43 (0.79,2.57) | 0.23 | 0.80 (0.50,1.26) | 0.34 | 1.06 (0.56,2.01) | 0.86 | 0.79 (0.48,1.30) | 0.36 |
| *>4 to ≤ 6 (most deprived)* | 0.64 (0.09,4.52) | 0.65 | 0.99 (0.62,1.58) | 0.98 | 1.09 (0.62,1.94) | 0.76 | 0.80 (0.50,1.27) | 0.35 | 1.15 (0.60,2.21) | 0.66 | 1.03 (0.61,1.73) | 0.92 |
| **About you** |  |  |  |  |  |  |  |  |  |  |  |  |
| **Diabetes Type** *Base: Type 2* |  |  |  |  |  |  |  |  |  |  |  |  |
| *Type 1* | 0.44 (0.04,4.39) | 0.49 | 0.76 (0.45,1.29) | 0.32 | 1.56 (0.74,3.32) | 0.25 | 1.39 (0.80,2.39) | 0.24 | 2.37 (1.22,4.61) | 0.01 | 1.15 (0.62,2.14) | 0.66 |
| *Other/don't know* | 0.31 (0.04,2.14) | 0.23 | 0.71 (0.38,1.30) | 0.26 | 0.93 (0.44,1.96) | 0.84 | 0.89 (0.48,1.66) | 0.71 | 2.03 (0.88,4.66) | 0.09 | 1.04 (0.53,2.01) | 0.92 |
| **Duration of diabetes**  *Base: 0-5 years* |  |  |  |  |  |  |  |  |  |  |  |  |
| *6 - 10 years* | 2.51 (0.41,15.40) | 0.32 | 0.95 (0.64,1.41) | 0.81 | 1.76 (1.05,2.93) | 0.03 | 1.39 (0.93,2.07) | 0.11 | 1.14 (0.65,2.03) | 0.64 | 1.12 (0.72,1.72) | 0.61 |
| *11 - 15 years* | 1.52 (0.21,10.83) | 0.68 | 1.35 (0.85,2.13) | 0.20 | 1.94 (1.07,3.52) | 0.03 | 1.73 (1.09,2.74) | 0.02 | 1.43 (0.77,2.66) | 0.26 | 1.06 (0.65,1.72) | 0.83 |
| *16+ years* | 5.35 (0.39,72.79) | 0.21 | 1.02 (0.66,1.57) | 0.94 | 1.05 (0.60,1.83) | 0.85 | 0.87 (0.56,1.34) | 0.53 | 1.09 (0.58,2.04) | 0.79 | 0.88 (0.54,1.42) | 0.60 |
| *missing* | 1.00 (1.00,1.00) | - | 2.02 (0.34,12.09) | 0.44 | 1.00 (1.00,1.00) | - | 1.00 (1.00,1.00) | - | 1.00 (1.00,1.00) | - | 1.31 (0.22,7.88) | 0.77 |
| **Last attended DESP**  *Base: Within last two years* |  |  |  |  |  |  |  |  |  |  |  |  |
| *NOT within last two years (including first time attender/do not remember/missing)* | 0.64 (0.12,3.44) | 0.60 | 1.06 (0.66,1.71) | 0.81 | 1.05 (0.60,1.85) | 0.86 | 1.04 (0.65,1.68) | 0.87 | 0.70 (0.33,1.49) | 0.35 | 1.08 (0.64,1.81) | 0.78 |
| **Trust in health-based results from used applications** *Base: Sometimes* |  |  |  |  |  |  |  |  |  |  |  |  |
| *Unsure, never or rarely* | 0.32 (0.04,2.59) | 0.29 | 0.68 (0.37,1.27) | 0.23 | 0.45 (0.23,0.88) | 0.02 | 0.85 (0.46,1.58) | 0.60 | 1.17 (0.52,2.60) | 0.71 | 0.89 (0.45,1.75) | 0.73 |
| *Often* | 1.18 (0.23,5.96) | 0.84 | 1.14 (0.81,1.62) | 0.45 | 1.58 (1.00,2.50) | 0.05 | 1.99 (1.40,2.83) | 1.24E^-04^ | 0.58 (0.34,0.97) | 0.04 | 1.39 (0.94,2.05) | 0.10 |
| *Very often or always* | 1.12 (0.14,9.25) | 0.92 | 1.70 (1.06,2.73) | 0.03 | 4.65 (2.09,10.33) | 1.63E^-04^ | 3.67 (2.18,6.18) | 1.06E^-06^ | 0.56 (0.26,1.23) | 0.15 | 1.48 (0.89,2.46) | 0.13 |

Q5: ''The Diabetic Eye Screening Programme is important in monitoring my condition.'

Q17: “'I am concerned that not knowing how AI works is a barrier to people living with diabetes accepting this technology for diabetic eye screening”

Q21: “If my eye images were processed by a computer or AI technology, I would be happy for these data to be securely stored under the control of the NHS and used for research to do with healthcare evaluation or improvement”

Q23: “I believe AI could detect diabetic eye disease equally well in people of different ethnic groups and different ages”

Q24_3_a: “It would be important for me to receive the results of my eye assessment on the day”

Q24_4_a: “I would be happy to receive results from my eye assessment via a text message or email rather than in a letter”

## Table S7: Multivariable Linear regression results among health app users (health care practitioners)

| **Differences in scores compared to base group (95% confidence interval)** | | | | | | | | | | | | | | |
| --- | --- | --- | --- | --- | --- | --- | --- | --- | --- | --- | --- | --- | --- | --- |
| **Characteristics** | **General score** | **p-value** | **Efficiency score** | **p-value** | **Data security score** | **p-value** | **Trust score** | **p-value** | **Workforce (+) score** | **p-value** | **Workforce (-) score** | **p-value** | **Screening experience** | **p-value** |
| **Site**  *Base: BARS* |  |  |  |  |  |  |  |  |  |  |  |  |  |  |
| *North East London* | 0.04  (-0.30,0.38) | 0.81 | 0.32  (-0.06,0.70) | 0.10 | -0.32  (-0.71,0.06) | 0.10 | 0.16  (-0.21,0.53) | 0.39 | 0.17  (-0.21,0.55) | 0.38 | -0.15  (-0.61,0.30) | 0.50 | 0.05  (-0.32,0.42) | 0.78 |
| *South East London* | -0.29  (-0.68,0.10) | 0.14 | 0.14  (-0.30,0.57) | 0.54 | -0.42  (-0.87,0.02) | 0.06 | -0.06  (-0.49,0.36) | 0.77 | 0.11  (-0.33,0.55) | 0.62 | -0.35  (-0.87,0.17) | 0.19 | -0.12  (-0.54,0.30) | 0.58 |
| *Gloucester* | 0.16  (-0.21,0.52) | 0.40 | 0.09  (-0.33,0.50) | 0.69 | -0.28  (-0.70,0.14) | 0.19 | 0.16  (-0.25,0.56) | 0.44 | 0.45  (0.03,0.86) | 0.03 | 0.04  (-0.45,0.53) | 0.87 | 0.14  (-0.26,0.54) | 0.49 |
| *Tyneside* | 0.26  (-0.19,0.72) | 0.26 | 0.40  (-0.12,0.92) | 0.13 | -0.54  (-1.07,-0.02) | 0.04 | 0.42  (-0.08,0.92) | 0.10 | 0.61  (0.10,1.13) | 0.02 | -0.17  (-0.78,0.45) | 0.59 | -0.02  (-0.52,0.48) | 0.93 |
| **Age group** *Base: ≥ 50 to < 60 years* |  |  |  |  |  |  |  |  |  |  |  |  |  |  |
| *< 30 years* | 0.40  (-0.01,0.81) | 0.05 | 0.44  (-0.02,0.90) | 0.06 | 0.35  (-0.11,0.82) | 0.13 | 0.22  (-0.22,0.67) | 0.32 | 0.20  (-0.26,0.65) | 0.40 | -0.12  (-0.67,0.42) | 0.66 | 0.03  (-0.41,0.47) | 0.90 |
| *≥ 30 to < 40 years* | 0.13  (-0.18,0.45) | 0.41 | 0.11  (-0.25,0.47) | 0.54 | 0.09  (-0.28,0.45) | 0.64 | -0.08  (-0.43,0.27) | 0.65 | -0.06  (-0.42,0.30) | 0.74 | -0.10  (-0.53,0.33) | 0.64 | -0.13  (-0.48,0.21) | 0.45 |
| *≥ 40 to < 50 years* | 0.21  (-0.10,0.51) | 0.18 | 0.01  (-0.33,0.35) | 0.95 | 0.38  (0.03,0.73) | 0.03 | -0.13  (-0.47,0.20) | 0.43 | -0.12  (-0.46,0.23) | 0.51 | 0.36  (-0.05,0.77) | 0.08 | 0.02  (-0.31,0.36) | 0.88 |
| *60+ years* | 0.58  (0.20,0.97) | 3.06E^-03^ | 0.32  (-0.12,0.76) | 0.15 | -0.10  (-0.54,0.34) | 0.65 | 0.34  (-0.09,0.76) | 0.12 | 0.18  (-0.25,0.62) | 0.41 | 0.57  (0.05,1.09) | 0.03 | 0.34  (-0.08,0.76) | 0.12 |
| **Sex** *Base: male* |  |  |  |  |  |  |  |  |  |  |  |  |  |  |
| *Female* | -0.28  (-0.52,-0.03) | 0.03 | -0.33  (-0.60,-0.05) | 0.02 | -0.21  (-0.48,0.07) | 0.15 | -0.26  (-0.53,0.01) | 0.06 | -0.29  (-0.56,-0.01) | 0.04 | -0.38  (-0.71,-0.05) | 0.02 | -0.21  (-0.48,0.06) | 0.12 |
| *Prefer not to say* | -1.06  (-2.06,-0.06) | 0.04 | -1.45  (-2.58,-0.31) | 0.01 | -1.02  (-2.16,0.13) | 0.08 | -1.34  (-2.44,-0.24) | 0.02 | -0.63  (-1.76,0.50) | 0.27 | -0.13  (-1.48,1.21) | 0.85 | -1.02  (-2.12,0.07) | 0.07 |
| **Ethnicity**  *Base: White* |  |  |  |  |  |  |  |  |  |  |  |  |  |  |
| *Black* | -0.15  (-0.86,0.56) | 0.68 | 0.32  (-0.49,1.13) | 0.44 | -0.62  (-1.43,0.20) | 0.14 | -0.01  (-0.79,0.78) | 0.98 | 0.63  (-0.17,1.44) | 0.12 | 0.16  (-0.80,1.12) | 0.74 | 0.16  (-0.62,0.94) | 0.69 |
| *Asian* | -0.54  (-0.89,-0.20) | 1.98E^-03^ | -0.42  (-0.81,-0.03) | 0.04 | -0.63  (-1.02,-0.24) | 1.68E^-03^ | -0.72  (-1.10,-0.34) | 1.93E^-04^ | -0.33  (-0.72,0.06) | 0.09 | -0.55  (-1.01,-0.08) | 0.02 | -0.41  (-0.79,-0.04) | 0.03 |
| *Mixed / other / prefer not to say* | 0.45  (0.02,0.87) | 0.04 | 0.22  (-0.26,0.70) | 0.36 | 0.03  (-0.45,0.52) | 0.89 | 0.38  (-0.09,0.84) | 0.11 | 0.20  (-0.28,0.68) | 0.41 | -0.03  (-0.59,0.54) | 0.93 | 0.21  (-0.25,0.68) | 0.37 |
| **Townsend score** *Base: ≥ -0.5 to < 2* |  |  |  |  |  |  |  |  |  |  |  |  |  |  |
| *<-2 (least deprived)* | -0.02  (-0.36,0.31) | 0.90 | 0.13  (-0.25,0.50) | 0.52 | -0.11  (-0.49,0.28) | 0.59 | -0.09  (-0.46,0.28) | 0.65 | 0.17  (-0.20,0.55) | 0.37 | -0.50  (-0.96,-0.05) | 0.03 | -0.07  (-0.44,0.29) | 0.69 |
| *≥ -2 to <-0.5* | -0.22  (-0.54,0.11) | 0.19 | -0.25  (-0.62,0.11) | 0.18 | -0.10  (-0.47,0.27) | 0.59 | -0.45  (-0.81,-0.09) | 0.01 | -0.26  (-0.63,0.11) | 0.16 | -0.44  (-0.88,0.00) | 0.05 | 0.04  (-0.32,0.40) | 0.82 |
| *≥ 2 to <4* | -0.12  (-0.46,0.23) | 0.50 | -0.29  (-0.68,0.10) | 0.14 | -0.05  (-0.44,0.35) | 0.81 | -0.34  (-0.72,0.04) | 0.08 | -0.11  (-0.50,0.28) | 0.58 | -0.17  (-0.63,0.29) | 0.47 | -0.11  (-0.49,0.27) | 0.57 |
| *≥ 4.0 (most deprived)* | 0.17  (-0.18,0.52) | 0.35 | -0.07  (-0.47,0.32) | 0.73 | -0.01  (-0.41,0.39) | 0.95 | -0.14  (-0.53,0.24) | 0.47 | 0.17  (-0.23,0.56) | 0.40 | -0.02  (-0.49,0.45) | 0.93 | -0.10  (-0.48,0.28) | 0.61 |
| *missing* | 0.39  (-0.40,1.19) | 0.33 | 0.42  (-0.47,1.32) | 0.36 | 0.04  (-0.87,0.95) | 0.93 | 0.93  (0.06,1.80) | 0.04 | 0.60  (-0.29,1.50) | 0.19 | -0.20  (-1.26,0.87) | 0.72 | 0.32  (-0.55,1.18) | 0.48 |
| **Role** *Base: Screener, grader, photographer or optometrist* |  |  |  |  |  |  |  |  |  |  |  |  |  |  |
| *Clinical Lead or management position* | 0.25  (-0.03,0.54) | 0.08 | 0.20  (-0.13,0.52) | 0.23 | -0.07  (-0.39,0.26) | 0.69 | 0.16  (-0.15,0.48) | 0.30 | 0.37  (0.05,0.69) | 0.02 | 0.24  (-0.14,0.62) | 0.22 | 0.23  (-0.08,0.54) | 0.15 |
| *Senior screener /grader* | -0.02  (-0.33,0.28) | 0.87 | -0.06  (-0.40,0.28) | 0.74 | 0.18  (-0.17,0.52) | 0.31 | -0.04  (-0.37,0.29) | 0.82 | 0.19  (-0.15,0.53) | 0.26 | 0.12  (-0.28,0.53) | 0.56 | 0.14  (-0.19,0.47) | 0.42 |
| *Administrator, fail safe officer, IT officer or other* | -0.02  (-0.37,0.33) | 0.91 | -0.26  (-0.66,0.13) | 0.19 | 0.30  (-0.09,0.70) | 0.13 | -0.02  (-0.40,0.36) | 0.92 | -0.05  (-0.44,0.34) | 0.81 | 0.15  (-0.32,0.62) | 0.53 | 0.13  (-0.25,0.51) | 0.50 |
| **Length in current role** *Base: Less than 5 years* |  |  |  |  |  |  |  |  |  |  |  |  |  |  |
| *5 to less than 10 years* | 0.04  (-0.24,0.31) | 0.79 | -0.09  (-0.41,0.22) | 0.56 | -0.04  (-0.36,0.27) | 0.79 | -0.05  (-0.35,0.26) | 0.77 | 0.05  (-0.26,0.36) | 0.76 | -0.08  (-0.45,0.29) | 0.67 | -0.16  (-0.46,0.14) | 0.29 |
| *10 years or more* | -0.26  (-0.55,0.02) | 0.07 | -0.33  (-0.65,-0.01) | 0.05 | 0.02  (-0.31,0.34) | 0.91 | -0.29  (-0.61,0.02) | 0.07 | -0.11  (-0.43,0.21) | 0.50 | -0.39  (-0.77,-0.01) | 0.04 | -0.34  (-0.65,-0.03) | 0.03 |
| **Technology in daily life** *Base: 5 or more applications* |  |  |  |  |  |  |  |  |  |  |  |  |  |  |
| *less than 5 applications* | -0.38  (-0.81,0.05) | 0.08 | -0.65  (-1.14,-0.16) | 0.01 | 0.53  (0.04,1.02) | 0.04 | -0.45  (-0.93,0.02) | 0.06 | -0.74  (-1.23,-0.25) | 2.86E^-03^ | 0.07  (-0.51,0.65) | 0.82 | 0.06  (-0.42,0.53) | 0.82 |
| **Trust in health-based results from used applications**  *Base: Sometimes* |  |  |  |  |  |  |  |  |  |  |  |  |  |  |
| *Unsure, never or rarely* | -0.40  (-0.78,-0.02) | 0.04 | -0.55  (-0.98,-0.12) | 0.01 | -0.03  (-0.47,0.41) | 0.89 | -0.41  (-0.83,0.01) | 0.06 | -0.61  (-1.05,-0.18) | 0.01 | 0.39  (-0.13,0.90) | 0.14 | -0.30  (-0.71,0.12) | 0.17 |
| *Often* | 0.41  (0.16,0.65) | 1.23E^-03^ | 0.37  (0.10,0.65) | 8.58E^-03^ | 0.25  (-0.03,0.53) | 0.08 | 0.48  (0.21,0.75) | 5.51E^-04^ | 0.19  (-0.09,0.47) | 0.19 | 0.46  (0.13,0.79) | 0.01 | 0.42  (0.15,0.69) | 2.20E^-03^ |
| *Very often or always* | 0.72  (0.26,1.17) | 2.08E^-03^ | 0.72  (0.20,1.23) | 6.44E^-03^ | 0.38  (-0.14,0.90) | 0.15 | 1.21  (0.70,1.71) | 2.58E^-06^ | 0.48  (-0.04,0.99) | 0.07 | 0.84  (0.23,1.46) | 6.98E^-03^ | 0.44  (-0.06,0.93) | 0.09 |

## Table S8: Multivariable Logistic regression results among health app users (health care practitioners)

| **Odds ratio for stand-alone questions (95% confidence interval) [HCP]** | | | | | | | | |
| --- | --- | --- | --- | --- | --- | --- | --- | --- |
| **Characteristic** | **Q11** | **p-value** | **Q15** | **p-value** | **Q16_5_a** | **p-value** | **Q18** | **p-value** |
| **Site** *Base: BARS* |  |  |  |  |  |  |  |  |
| *North East London* | 0.45 (0.15,1.42) | 0.18 | 0.44 (0.10,1.93) | 0.28 | 1.16 (0.18,7.50) | 0.87 | 1.40 (0.40,4.94) | 0.60 |
| *South East London* | 6.58 (1.16,37.33) | 0.03 | 0.97 (0.19,4.92) | 0.97 | 2.93 (0.25,34.30) | 0.39 | 0.53 (0.13,2.19) | 0.38 |
| *Gloucester* | 1.55 (0.39,6.14) | 0.53 | 0.15 (0.03,0.78) | 0.02 | 1.34 (0.21,8.43) | 0.76 | 2.00 (0.41,9.87) | 0.39 |
| *Tyneside* | 1.28 (0.28,5.91) | 0.75 | 1.07 (0.13,9.10) | 0.95 | 17.08 (1.58,184.46) | 0.02 | 1.00 (1.00,1.00) | - |
| **Age group** *Base: ≥ 50 to < 60 years* |  |  |  |  |  |  |  |  |
| *< 30 years* | 1.94 (0.44,8.56) | 0.38 | 18.23 (2.48,134.11) | 4.35E^-03^ | 0.13 (0.01,1.12) | 0.06 | 0.39 (0.07,2.07) | 0.27 |
| *≥ 30 to < 40 years* | 1.41 (0.45,4.41) | 0.55 | 2.55 (0.63,10.34) | 0.19 | 0.41 (0.07,2.39) | 0.32 | 0.86 (0.23,3.23) | 0.82 |
| *≥ 40 to < 50 years* | 0.63 (0.22,1.80) | 0.39 | 2.47 (0.65,9.49) | 0.19 | 0.55 (0.10,3.02) | 0.49 | 0.25 (0.07,0.87) | 0.03 |
| *60+ years* | 0.56 (0.15,2.06) | 0.39 | 22.23 (3.90,126.82) | 4.80E^-04^ | 0.59 (0.07,4.63) | 0.61 | 0.51 (0.10,2.51) | 0.40 |
| **Sex** *Base: Male* |  |  |  |  |  |  |  |  |
| *Female* | 0.80 (0.34,1.88) | 0.61 | 0.55 (0.20,1.52) | 0.25 | 1.26 (0.31,5.03) | 0.75 | 0.47 (0.16,1.34) | 0.16 |
| *Prefer not to say* | 0.41 (0.01,14.63) | 0.63 | 1.00 (1.00,1.00) | - | 1.00 (1.00,1.00) | - | 0.21 (0.01,6.17) | 0.37 |
| **Ethnicity** *Base: White* |  |  |  |  |  |  |  |  |
| *Black* | 1.00 (1.00,1.00) | - | 1.05 (0.04,26.69) | 0.98 | 17.57 (1.18,261.83) | 0.04 | 2.01 (0.12,34.57) | 0.63 |
| *Asian* | 1.92 (0.57,6.52) | 0.29 | 0.16 (0.03,0.84) | 0.03 | 2.60 (0.43,15.68) | 0.30 | 1.29 (0.37,4.51) | 0.69 |
| *Mixed/other/prefer not to say* | 1.00 (0.23,4.43) | 1.00 | 2.64 (0.45,15.60) | 0.28 | 0.58 (0.04,8.25) | 0.69 | 3.45 (0.49,24.50) | 0.22 |
| **Townsend score** *Base: ≥ -0.5 to <2* |  |  |  |  |  |  |  |  |
| *<-2 (least deprived)* | 2.02 (0.64,6.40) | 0.23 | 1.42 (0.36,5.59) | 0.62 | 3.87 (0.63,23.75) | 0.14 | 1.08 (0.29,4.04) | 0.91 |
| *≥ -2 to <-0.5* | 2.15 (0.69,6.71) | 0.19 | 0.38 (0.08,1.79) | 0.22 | 3.76 (0.68,20.80) | 0.13 | 1.12 (0.27,4.70) | 0.87 |
| *≥ 2 to <4* | 1.57 (0.49,5.03) | 0.45 | 2.69 (0.65,11.12) | 0.17 | 1.13 (0.13,10.13) | 0.91 | 0.36 (0.10,1.31) | 0.12 |
| *≥ 4.0 (most deprived)* | 1.61 (0.46,5.67) | 0.46 | 4.08 (0.92,18.14) | 0.06 | 0.84 (0.09,8.22) | 0.88 | 0.37 (0.10,1.45) | 0.15 |
| *missing* | 3.44 (0.22,54.26) | 0.38 | 0.39 (0.02,8.87) | 0.55 | 32.81 (1.13,952.64) | 0.04 | 1.00 (1.00,1.00) | - |
| **Role** *Base: Screener, grader, photographer or optometrist* |  |  |  |  |  |  |  |  |
| *Clinical Lead or management position* | 0.76 (0.28,2.05) | 0.59 | 1.25 (0.39,3.98) | 0.71 | 0.10 (0.02,0.68) | 0.02 | 0.63 (0.21,1.86) | 0.40 |
| *Senior screener /grader* | 0.49 (0.17,1.40) | 0.18 | 0.15 (0.03,0.73) | 0.02 | 0.40 (0.08,1.95) | 0.26 | 0.72 (0.22,2.30) | 0.58 |
| *Administrator, fail safe officer, IT officer or other* | 0.52 (0.15,1.77) | 0.30 | 1.28 (0.31,5.20) | 0.73 | 0.02 (0.00,0.33) | 0.01 | 0.89 (0.20,4.06) | 0.89 |
| **Length of role**  *Base: Less than 5 years* |  |  |  |  |  |  |  |  |
| *5 to less than 10 years* | 2.01 (0.74,5.43) | 0.17 | 1.61 (0.49,5.24) | 0.43 | 0.49 (0.12,1.93) | 0.31 | 0.90 (0.31,2.63) | 0.85 |
| *10 years or more* | 1.99 (0.75,5.30) | 0.17 | 1.05 (0.33,3.38) | 0.94 | 0.19 (0.04,0.95) | 0.04 | 0.80 (0.26,2.46) | 0.70 |
| **Technology in daily life** *Base: 5 or more applications* |  |  |  |  |  |  |  |  |
| *less than 5 applications* | 0.50 (0.11,2.24) | 0.36 | 0.43 (0.05,3.63) | 0.44 | 2.12 (0.27,16.76) | 0.48 | 0.83 (0.15,4.64) | 0.83 |
| T**rust in health-based results from used applications** *Base: Sometimes* |  |  |  |  |  |  |  |  |
| *Unsure, never or rarely* | 0.38 (0.10,1.50) | 0.17 | 0.09 (0.01,1.07) | 0.06 | 4.79 (0.92,24.82) | 0.06 | 1.29 (0.26,6.39) | 0.76 |
| *Often* | 0.59 (0.25,1.39) | 0.23 | 3.50 (1.31,9.35) | 0.01 | 1.67 (0.47,5.97) | 0.43 | 0.95 (0.37,2.48) | 0.92 |
| *Very often or always* | 0.64 (0.14,3.01) | 0.57 | 27.15 (3.56,207.02) | 1.45E^-03^ | 1.00 (1.00,1.00) | - | 4.66 (0.34,64.58) | 0.25 |

Q11: “A lack of transparency in how AI works is a barrier to health care professionals accepting this technology for use within the DESP”

Q15: “I believe AI could detect diabetic eye disease equally well in people of different ethnic groups and different ages”

Q16_5_a: “...wholly replace human grading of retinal images for detection of diabetic eye disease within the DESP”

Q18: “Further training would be required for staff working in the DESP if AI systems for analysing retinal images were to be implemented”

## Text S2: Additional References

31. Willis K, Chaudhry UAR, Chandrasekaran L et al. What are the perceptions and concerns of people living with diabetes and National Health Service staff around the potential implementation of AI-assisted screening for diabetic eye disease? Development and validation of a survey for use in a secondary care setting. BMJ Open. 2023;13(11):e075558. doi: 10.1136/bmjopen-2023-075558.

32. Jutzi TB, Krieghoff-Henning EI, Holland-Letz T et al. Artificial Intelligence in Skin Cancer Diagnostics: The Patients' Perspective. Front Med. 2020;7:233. doi: 10.3389/fmed.2020.00233

33. Lennox-Chhugani N, Chen Y, Pearson V, Trzcinski B, James J. Women’s attitudes to the use of AI image readers: a case study from a national breast screening programme. BMJ Health Care Inform. 2021;28(1):e100293. doi: 10.1136/bmjhci-2020-100293

34. Thornton N, Binesmael A, Horton T, Hardie T. AI in health care: what do the public and NHS staff think? 2024. Available from: https://www.health.org.uk/publications/long-reads/ai-in-health-care-what-do-the-public-and-nhs-staff-think. Accessed 6 August 2024

35. Fazakarley CA, Breen M, Leeson P, Thompson B, Williamson V. Experiences of using artificial intelligence in healthcare: a qualitative study of UK clinician and key stakeholder perspectives. BMJ Open. 2023;13(12):e076950. doi: 10.1136/bmjopen-2023-076950

36. Witkowski K, Okhai R, Neely SR. Public perceptions of artificial intelligence in healthcare: ethical concerns and opportunities for patient-centered care. BMC Med Ethics. 2024;25(1):74. doi: 10.1186/s12910-024-01066-4

37. Musbahi O, Syed L, Le Feuvre P, Cobb J, Jones G. Public patient views of artificial intelligence in healthcare: A nominal group technique study. Digit Health. 2021;7:20552076211063682. doi: 10.1177/20552076211063682

38. NHS Transformation Directorate. Artificial Intelligence. Available from: https://transform.england.nhs.uk/information-governance/guidance/artificial-intelligence/. Accessed 10 October 2025.

39. Wahlich C, Chandrasekaran L, Chaudhry UAR et al. Patient and practitioner perceptions around use of artificial intelligence within the English NHS diabetic eye screening programme. Diabetes Res Clin Pract. 2025;219:111964. doi: 10.1016/j.diabres.2024.111964

40. Mehta N, Harish V, Bilimoria K *et al.* Knowledge and Attitudes on Artificial Intelligence in Healthcare: A Provincial Survey Study of Medical Students [version 1]. MedEdPublish 2021, **10**:75 (<https://doi.org/10.15694/mep.2021.000075.1>)

41. Young AT, Amara D, Bhattacharya A, Wei ML. Patient and general public attitudes towards clinical artificial intelligence: a mixed methods systematic review

The Lancet Digital Health, Volume 3, Issue 9, e599 - e611

42. Jebb AT, Ng V, Tay L. A Review of Key Likert Scale Development Advances: 1995–2019. Front Psychol. 2021;12. doi: 10.3389/fpsyg.2021.637547
